# Activation of innate-adaptive immune machinery by poly(I:C) exposes a therapeutic vulnerability to prevent relapse in stroma-rich colon cancer

**DOI:** 10.1136/gutjnl-2021-326183

**Published:** 2022-04-27

**Authors:** Shania M Corry, Amy MB McCorry, Tamsin RM Lannagan, Niamh A Leonard, Natalie C Fisher, Ryan M Byrne, Petros Tsantoulis, Xabier Cortes-Lavaud, Raheleh Amirkhah, Keara L Redmond, Aoife J McCooey, Sudhir B Malla, Emily Rogan, Svetlana Sakhnevych, Michael A Gillespie, Mark White, Susan D Richman, Rene-Filip Jackstadt, Andrew D Campbell, Sarah Maguire, S:CORT and ACRCelerate consortia, Simon S McDade, Daniel B Longley, Maurice B Loughrey, Helen G Coleman, Emma M Kerr, Sabine Tejpar, Timothy Maughan, Simon J Leedham, Donna M Small, Aideen E Ryan, Owen J Sansom, Mark Lawler, Philip D Dunne

**Affiliations:** 1 Patrick G Johnston Centre for Cancer Research, Queen's University Belfast, Belfast, UK; 2 Cancer Research UK, Beatson Institute for Cancer Research, Glasgow, UK; 3 Lambe Institute for Translational Research, College of Medicine Nursing and Health Sciences, National University of Ireland, Galway, Ireland; 4 Discipline of Pharmacology & Therapeutics, School of Medicine, National University of Ireland, Galway, Ireland; 5 Université de Genève, Geneva, Switzerland; 6 Institute of Cancer Sciences, University of Glasgow, Glasgow, UK; 7 Leeds Institute of Medical Research, University of Leeds, Leeds, UK; 8 Heidelberg Institute for Stem Cell Technology and Experimental Medicine (HI-STEM gGmbH) and Cancer Progression and Metastasis Group, German Cancer Research Center (DKFZ), Heidelberg, Germany; 9 Cellular Pathology, Belfast Health and Social Care Trust, Belfast, UK; 10 Centre for Public Health, Queens University Belfast, Belfast, UK; 11 Digestive Oncology Unit, University Ospital Gasthuisberg, Leuven, Belgium; 12 Department of Oncology, University of Oxford, Oxford, UK; 13 Wellcome Trust Centre Human Genetics, University of Oxford, Oxford, UK

**Keywords:** CANCER, COLON CARCINOGENESIS, COLORECTAL CANCER, ADJUVANT TREATMENT

## Abstract

**Objective:**

Stroma-rich tumours represent a poor prognostic subtype in stage II/III colon cancer (CC), with high relapse rates and limited response to standard adjuvant chemotherapy.

**Design:**

To address the lack of efficacious therapeutic options for patients with stroma-rich CC, we stratified our human tumour cohorts according to stromal content, enabling identification of the biology underpinning relapse and potential therapeutic vulnerabilities specifically within stroma-rich tumours that could be exploited clinically. Following human tumour-based discovery and independent clinical validation, we use a series of *in vitro* and stroma-rich *in vivo* models to test and validate the therapeutic potential of elevating the biology associated with reduced relapse in human tumours.

**Results:**

By performing our analyses specifically within the stroma-rich/high-fibroblast (HiFi) subtype of CC, we identify and validate the clinical value of a HiFi-specific prognostic signature (HPS), which stratifies tumours based on STAT1-related signalling (High-HPS v Low-HPS=HR 0.093, CI 0.019 to 0.466). Using *in silico, in vitro* and *in vivo* models, we demonstrate that the HPS is associated with antigen processing and presentation within discrete immune lineages in stroma-rich CC, downstream of double-stranded RNA and viral response signalling. Treatment with the TLR3 agonist poly(I:C) elevated the HPS signalling and antigen processing phenotype across *in vitro* and *in vivo* models. In an *in vivo* model of stroma-rich CC, poly(I:C) treatment significantly increased systemic cytotoxic T cell activity (p<0.05) and reduced liver metastases (p<0.0002).

**Conclusion:**

This study reveals new biological insight that offers a novel therapeutic option to reduce relapse rates in patients with the worst prognosis CC.

**Significance of this study**

**What is already known on this subject?**

- Stroma-rich tumour composition is associated with poor prognosis in patients with stage II/III colon cancer (CC), with a relapse rate of approximately 50% in this setting, even when patients are treated with standard adjuvant chemotherapy. Elevation of transforming growth factor-β (TGF-β) signalling is observed in stroma-rich CCs, which has been used as the basis for trials based on TGF-β blockade.

**What are the new findings?**

- In this study, we push beyond the established association between stromal-derived TGF-β and poor prognosis, to identify, characterise and therapeutically exploit the biology that underpins relapse specifically within this TGF-β-high poor prognostic group. This stroma-rich subtype-specific approach reveals that STAT1-mediated antigen processing and viral response signalling is a targetable therapeutic vulnerability, via toll-like receptor 3 (TLR3) agonist poly(I:C), specifically in stroma-rich CC.

**How might it impact on clinical practice in the foreseeable future?**

- This study reveals a new insight into the biology underpinning relapse in stroma-rich tumours, and offers a novel therapeutic option to reduce relapse rates in patients with stroma-rich tumours, which represents the worst prognostic subgroup in early stage CC.

## Introduction

Colorectal cancer (CRC) is the third most commonly diagnosed cancer worldwide, with around 1.3 million cases diagnosed each year.^1^ Despite improvements in both surgical management and adjuvant treatment options, many stage II and III colon cancer (CC) patients still experience relapse following surgery; ~20% and 36% of patients within each stage respectively.^2^ Classification of CRC patients into molecular subtypes, based on their underlying transcriptional signalling, revealed four consensus molecular subtypes (CMS1-4), where the stromal subtype (CMS4)^3^ has the most dismal prognosis. Alongside molecular subtyping, these poor prognostic stroma-rich tumours can be also be identified using histology.^4–7^ Based on this evidence, the stroma-rich or high-fibroblast subtype (HiFi) represents a poor prognostic subgroup in stage II/III CC, with relapse rates of ~50%–60%.^3 8^ Importantly, this poor prognosis remains an issue even when stroma-rich patients receive adjuvant treatment following surgery; limited benefits from FOLFOX (bolus and infused fluorouracil with oxaliplatin) and capecitabine with oxaliplatin regimes were observed in patients with stroma-rich tumours in the short course oncology therapy (SCOT) clinical trial^9^ and in a recent meta-analysis where adjuvant chemotherapy was found not to be effective in CMS4 tumours.^10^

Numerous studies, including our own, have defined and characterised the biology underpinning stromal-rich tumours compared with epithelium-rich (stromal-low) tumours, which is dominated by elevated transforming growth factor-β (TGF-β) signalling or other markers of mesenchymal/CMS4 biology.^11–14^ Elevation of TGF-β and stromal signalling cascades have been proposed as targets themselves, however no evidence has been shown that such biology is driving the differential outcomes in the ~50%–60% of stroma-rich tumours that eventually relapse, compared with those that do not. Identification and understanding of the biology underpinning disease relapse specifically within stroma-rich tumours, rather than simply the characteristics of stroma-rich vs stroma-low tumours, could be used to develop novel therapeutic interventions specifically for patients with stroma-rich/CMS4 tumours that relapse following surgery.

To elucidate biology associated with patient outcome in the stroma-rich histological subtype, we combined fibroblast stratification with supervised transcriptomic analysis, based on risk of relapse, to uncover biology of specific relevance in stroma-rich localised (stage II/III) CC. To exploit this new understanding, we performed a series of in silico analyses to identify potential molecular vulnerabilities and a therapeutic candidate. Using a number of in vitro and in vivo models, we tested and validated the functional significance and potential clinical utility of poly(I:C) as a subtype-specific treatment option aimed at preventing metastatic relapse specifically within stroma-rich CC.

## Results

### Prognostic value of morpho-molecular fibroblast measurement in tumour samples

**Figure 1:**
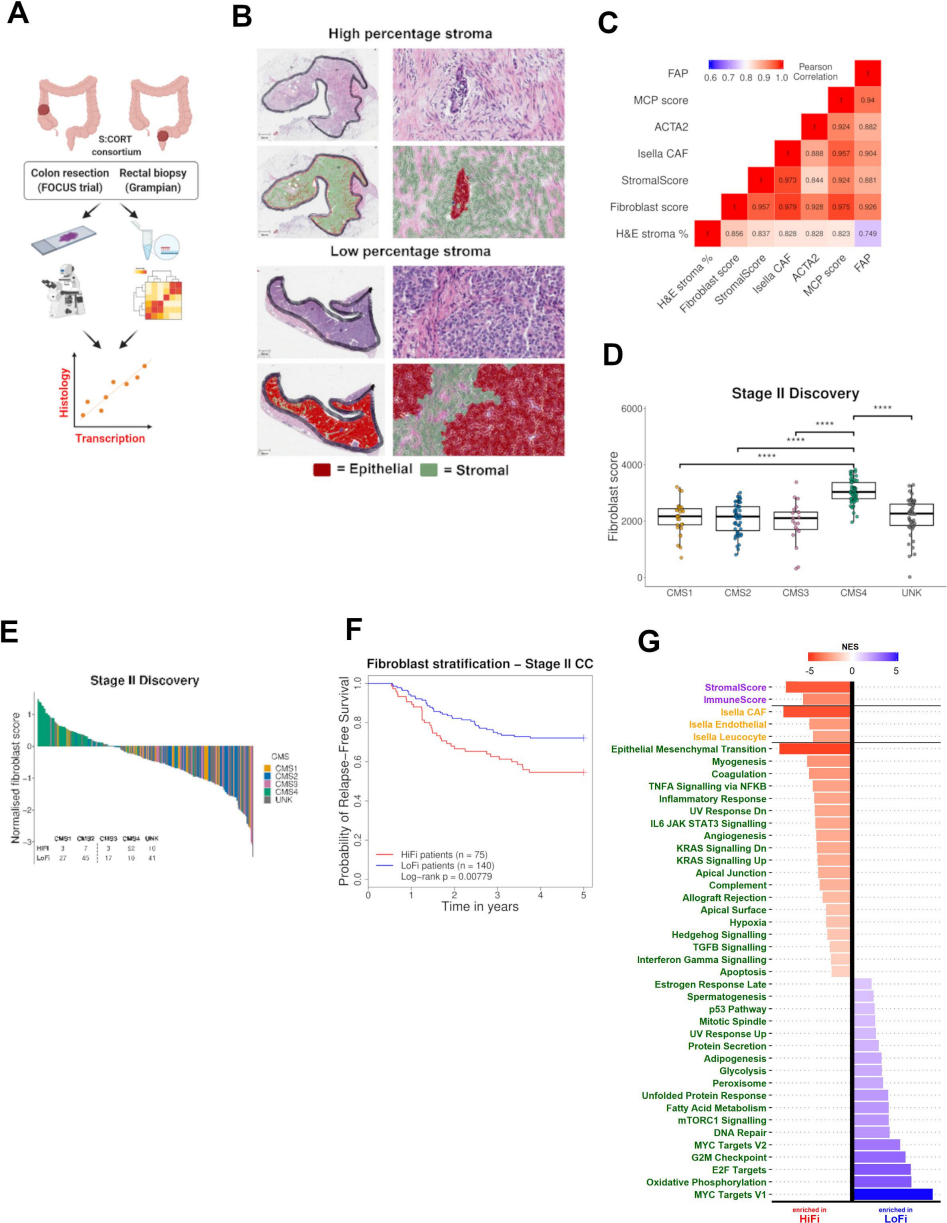
Development and validation of our transcriptional fibroblast score. (A) Schematic of correlation between stromal/fibroblasts scores via histology and transcriptomics. (B) H&E slide with the digital pathology stromal classifier applied to a sample with a high/low percentage stroma from the focus cohort. (C) Correlation matrix with histological stroma and transcriptional classifiers (Pearson’s correlation). (D) CMS classification according to our fibroblast score. (UNK=unknown/mixed CMS classification) (t-test). (E) Waterfall plot of fibroblast scores indicating CMS classification. High-fibroblast (HiFi) n=75 and low-fibroblast (LoFi) n=140. (F) HiFi tumours have a worse prognosis than LoFi in discovery cohort (log-rank p=0.00779) (G). Comparison of HiFi and LoFi samples revealed that previously published stromal signatures and gene sets have significantly higher expression in the HiFi samples than the LoFi (adjusted p<0.15). CC, colon cancer; CMS, consensus molecular subtypes. **** denotes p<0.0001.

To test the overlap between stromal gene signatures and histology, we used patient-matched transcriptional data and a QuPath^15^-derived H&E stromal classifier from colon resections (FOCUS cohort, n=361) and rectal pretreatment biopsies (Grampian cohort; n=225), previously characterised within the S:CORT stratified CRC programme^16^ (figure 1A). Strong correlations were observed between H&E digital stroma scores and a number of previously established transcriptional signatures, including StromalScore using ESTIMATE,^17^ cancer-associated fibroblast (CAF) score from Isella *et al* 18 and fibroblast score from MCPcounter,^19^ alongside individual CAF markers *ACTA2* (alpha-smooth muscle actin; αSMA) and FAP (figure 1B, C). Combining *ACTA2* and *FAP* gene expression with the existing MCP fibroblast signature generated a single-sample gene set enrichment analysis (ssGSEA) ‘fibroblast score’ transcriptional classifier, with a correlation higher than other methods (figure 1C; Pearson correlation=0.856, online supplemental table 1). This approach enabled us to employ transcriptional data from cohorts where no H&E images are available, with the understanding that our findings can be translatable to the stroma-rich histological subtype, traditionally identifiable from patient-matched H&E slides.

10.1136/gutjnl-2021-326183.supp1 Supplementary data

Using transcriptional data from a discovery cohort of n=215 untreated stage II CC tumours^20^ (online supplemental table 2), our ssGSEA fibroblast score was significantly higher in CMS4 tumours, compared with the other subtypes (figure 1D; t-test p<0.0001 for all). As fibroblast content is an already well-established prognostic biomarker, we defined an optimum prognostic cut-off level for our ssGSEA fibroblast scores, using relapse-free survival (RFS) data and Cox modelling (online supplemental figure 1). This resulted in stratification of the n=215 patients into high-fibroblast (HiFi; n=75; 35% of cohort) and low-fibroblast (LoFi; n=140; 65% of cohort) subgroups, with a larger proportion of HiFi tumours classified as CMS4 compared with LoFi tumours (69.1% and 7.1% respectively; figure 1E; Fisher’s exact test p<2.2×10^−16^, (online supplemental figure 1). The epithelium-rich CMS2 subtype is more prevalent in the LoFi group compared with HiFi (32.1% and 9.3% respectively; figure 1E; Fisher’s exact test p=2.28×10^−06^, (online supplemental figure 1). In line with previous studies, we observed significantly worse outcome in HiFi tumours compared with LoFi tumours, with patient relapse rates of 45.3% and 27.9%, respectively (figure 1F; log-rank p=0.00779, HR= 1.851, 95% CI (1.168 to 2.932)). In agreement with our initial correlative analyses (figure 1C), HiFi tumours also had significantly higher StromalScore using the ESTIMATE geneset, and fibroblast scores compared with LoFi tumours (figure 1G; adjusted p<0.15), alongside gene sets that we have previously directly associated with CAF infiltration,^21^ including the epithelial to mesenchymal transition (figure 1G).

### A number of previously identified prognostic factors are not prognostic in HiFi tumours

**Figure 2:**
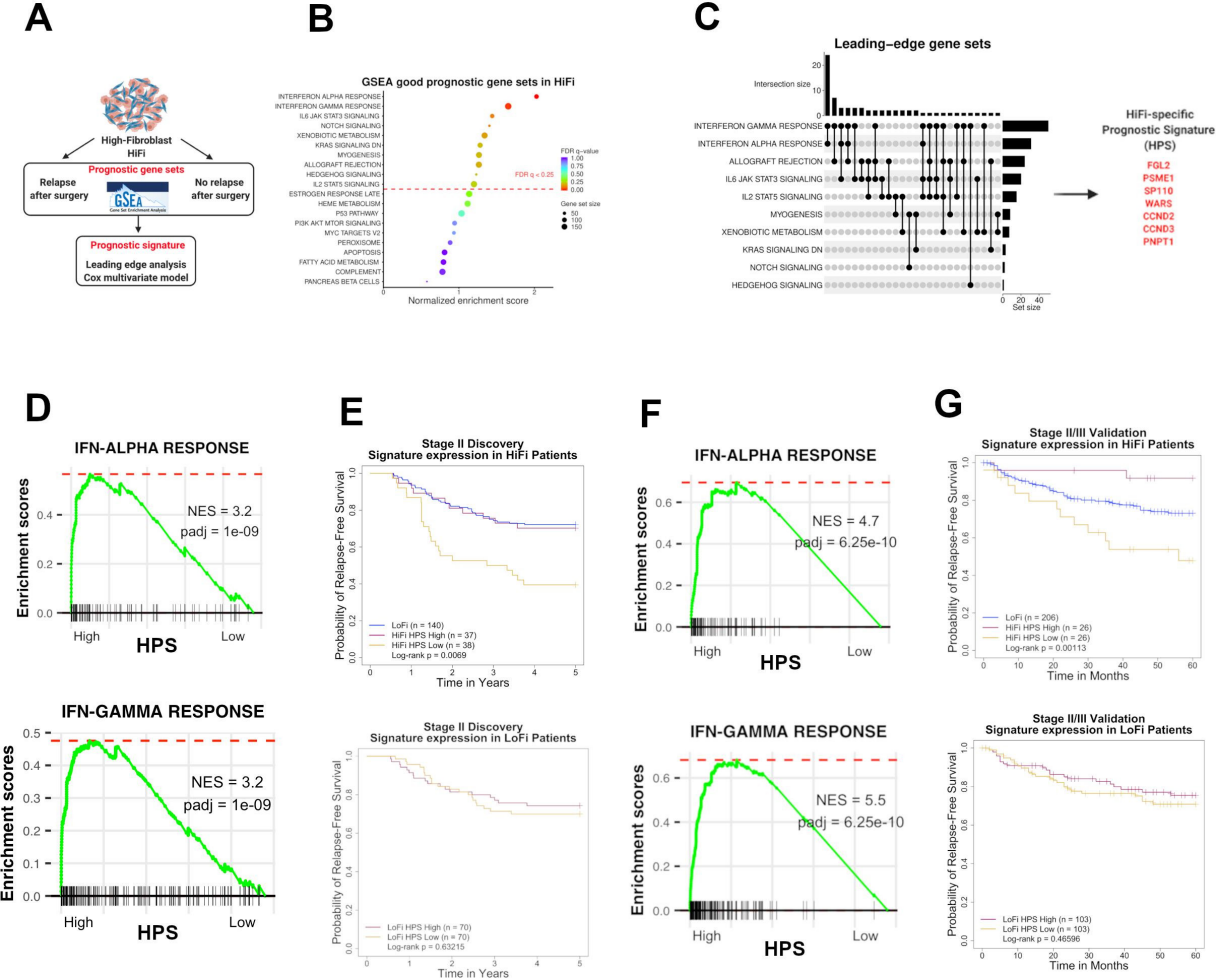
Identification of HiFi-specific prognostic biology (A) workflow summary of our supervised analyses. (B) Significant gene sets associated with good prognosis specifically within HiFi tumours from supervised GSEA analysis. (C) Leading-edge analysis (LEA) of the 10 gene sets demonstrating that, of the 71 genes, many of them overlap between the interferon response gene sets leading to identification of a seven gene HPS. (D) High expression of the HPS in HiFi tumours is associated with enriched IFN alpha and gamma response signalling in discovery cohort. (E) HPS has a strong prognostic value in HiFi tumours based on a median split in discovery cohort (log-rank p=0.0069; top). HPS has no prognostic value in the LoFi samples in discovery cohort (log-rank p=0.63215; bottom). (F) High expression of the HPS (n=26) in HIFI tumours is associated with enriched IFN alpha and gamma response signalling. (G) HPS can stratify HiFi samples into two groups in the validation cohort, one with significantly poorer RFS and another with RFS even better than the LoFi patients (log-rank p=0.00113; top). HPS has no prognostic value in the LoFi samples (log-rank p=0.46596; bottom). HiFi, high-fibroblast; LOFI, low-fibroblast; RFS, relapse-free survival.

Although HiFi tumours in our discovery cohort have a significantly worse prognosis compared with LoFi, the relapse rates in the HiFi subgroup remain ~40%–50% (figure 1F), meaning that approximately half of patients with stage II stroma-rich tumours are cured by surgery alone. In line with previous studies, we demonstrate the ability of TGF-β signalling, as assessed using a number of transcriptional signatures, to identify the stroma-rich subtype (online supplemental figure 2A). Importantly, however, when the prognostic value of these signatures are assessed specifically within the HiFi subtype, they do not stratify patients based on relapse status online supplemental figure 2B). Assessment of previously defined CAF subtypes developed in CC and pancreatic cancer^22–24^ failed to discriminate HiFi tumours based on relapse (online supplemental figure 2C); CRC CAF-A and CAF-B (upper left), pancreatic myCAF and iCAF (upper right), CRC differential contractility (lower left) and inflammatory-related fibroblasts CD34^-^THY1^+^, CD34^-^THY1^-^ and CD34^+^ CAF (lower right)). Similarly, stratification based on fibroblast stiffness-related matrix index,^25^ p53 activity (Hallmark gene set ssGSEA), stem-like markers, or overall fibroblast levels according to our ssGSEA score (online supplemental figure 2D) all failed to segregate the HiFi relapse and non-relapse tumours. Moreover, while unsupervised clustering of HiFi tumours identified two clusters, these subgroups did not have different prognostic outcomes (online supplemental figure 2E). As previously identified prognostic factors and unsupervised clustering provided no additional clinical value for identifying HiFi patients that relapse, we next performed a supervised analysis of tumours in the stage II untreated discovery cohort, using GSEA followed by leading-edge analysis (LEA) and Cox survival modelling, contrasting HiFi patients that relapsed within 5 years of surgery (n=34) and HiFi patients who never experienced disease relapse (n=41) (figure 2A).

### An interferon-related seven-gene signature identifies HiFi patients with significantly better prognosis

GSEA revealed 10 significant gene sets associated with good prognosis in the HiFi group, including elevated interferon alpha and interferon gamma response (figure 2B, (online supplemental figure 2F). Using a LEA, which reveals specific genes that contribute most to the gene sets associated with prognosis in HiFi tumours, we identified 71 genes shared by more than one of the LEA subsets (figure 2C; left). Cox survival analysis, followed by a multivariate model for each individual gene (to adjust for age, gender, pT stage, tumour location, tumour differentiation grade, lymphovascular invasion status and mucinous/non-mucinous subtype) filtered this list to seven LEA genes (p<0.05; table 1); namely *FGL2, PSME1, SP110, WARS, CCND2, CCND3, PNPT1,* which we term hereafter as a HiFi-specific prognostic signature (HPS) capable of distinguishing relapse from non-relapse (figure 2C; right). We next confirmed that stratification of HiFi patients using a median split of the HPS was sufficient to represent the same elevated interferon (IFN) alpha and IFN gamma response GSEA signatures (figure 2D), however HPS was not associated with the levels of TGF-β signalling in HiFi tumours (online supplemental figure 2G). This HPS median split was closely aligned to an area under the receiver operating characteristic (AUROC) optimal cut-off (online supplemental figure 3), which could significantly stratify patients with HiFi tumours based on relapse, where lower expression was associated with reduced RFS (figure 2E; top; log-rank p=0.0069) and those with high expression of HPS genes displayed RFS outcomes similar to those of LoFi patients.

**Table 1:** HiFi-specific prognostic signature and relapse free survival

Gene (median exp)	Univariate HR High versus Low (95% CI)	Univariate p value	Multivariate HR High versus Low (95% CI)	Multivariate p value	Total cases (relapse)	High exp (relapse)	Low exp (relapse)
FGL2 (5.497)	0.568 (0.282 to 1.142)	0.113	0.374 (0.166 to 0.840)	0.017	74 (33)	37 (13)	37 (20)
PSME1 (7.384)	0.541 (0.269 to 1.089)	0.085	0.364 (0.153 to 0.868)	0.023	74 (33)	37 (13)	37 (20)
SP110 (6.082)	0.498 (0.245 to 1.014)	0.055	0.339 (0.148 to 0.775)	0.010	74 (33)	37 (12)	37 (21)
WARS (6.510)	0.387 (0.187 to 0.801)	0.010	0.319 (0.145 to 0.705)	0.005	74 (33)	37 (33)	37 (20)
CCND2 (9.464)	0.574 (0.285 to 1.155)	0.120	0.438 (0.204 to 0.939)	0.034	74 (33)	37 (13)	37 (20)
CCND3 (7.715)	0.513 (0.252 to 1.043)	0.065	0.414 (0.192 to 0.893)	0.025	74 (33)	37 (12)	37 (21)
PNPT1 (2.560)	0.312 (0.148 to 0.657)	0.002	0.265 (0.118 to 0.593)	0.001	74 (33)	37 (10)	37 (23)

Dataset (median sig)	Univariate HR High versus CI)	Univariate p value	Multivariate HR High versus Low (95% CI)	Multivariate p value	Total cases (relapse)	High sig (relapse)	Low sig (relapse)
Discovery (6.482)	0.395 (0.191 to 0.816)	0.012	0.218 (0.087 to 0.544)	0.001	74 (33)	36 (10)	38 (23)
Validation (9.137)	0.123 (0.027 to 0.550)	0.006	0.093 (0.019 to 0.466)	0.004	52 (14)	26 (2)	26 (12)

Median gene expression values were used to dichotomise the HiFi patients into high and low expression groups in Discovery cohort. Multivariate Cox regression analysis adjusted for age, sex, pT stage, tumour location, tumour differentiation grade, tumour subtype (mucinous/non-mucinous), lymphovascular invasion and the number of lymph nodes with relapse free survival as the outcome variable.

Median signature expression was used to dichotomise the HiFi patients in each cohort into high and low expression groups in both Discovery and Validation cohorts. For the discovery cohort, the multivariate Cox regression analysis adjusted for age, sex, pT stage, tumour location, tumour differentiation grade, tumour subtype (mucinous/non-mucinous), lymphovascular invasion and the number of lymph nodes with relapse free survival as the outcome variable. For the validation cohort, the multivariate Cox regression analysis adjusted for age, sex, TNM stage and tumour location with relapse free survival as the outcome variable.

HiFi, high fibroblast.

The HPS was prognostic in the discovery cohort using either univariate (HR 0.395, 95% CI (0.191 to 0.816), Wald test p=0.012; table 1) or multivariate analysis adjusting for age, gender, tumour location, tumour differentiation, lymphovascular invasion status, tumour subtype and the number of lymph nodes (HR 0.218, 95% CI (0.087 to 0.544), Wald test p=0.001; table 1). Additionally, the prognostic value of the HPS was subtype-specific for patients with HiFi tumours, as it had no significant prognostic value when it was used to stratify patients with LoFi tumours (figure 2E; bottom; log-rank p=0.63215).

To independently validate these findings, we applied our ssGSEA fibroblast scoring method to transcriptional profiles from an independent validation cohort of untreated stage II/III CC tumours^26^ (GSE39582; n=258 (online supplemental table 3). Similar to the discovery cohort, ssGSEA fibroblast scores were significantly higher in the CMS4 tumours compared with all other subtypes (online supplemental figure 4A); t-test p<0.0001). In line with stroma-rich populations identified in publicly-available cohorts (online supplemental figure 4B), patients within the top 20% ssGSEA fibroblast score were classed as HiFi (n=52) and the remaining 80% classed as LoFi (n=206), where HiFi samples were largely, but not exclusively, CMS4 (online supplemental figure 4C). Conversely, LoFi samples predominantly consisted of epithelium-rich subtypes; CMS2 and CMS3 (online supplemental figure 4C, D). HiFi tumours displayed higher StromalScore, and higher fibroblast score, alongside an analogous pattern of enrichment to that of the discovery cohort (online supplemental figure 4E). Importantly, and in line with the discovery findings, stratification of the HiFi tumours in this independent validation cohort using the median of HPS (again closely aligned to AUROC optimal cut-off; online supplemental figure 3), revealed that those with a low expression (n=26) had significantly lower IFN alpha and IFN gamma response signalling (figure 2F) and poorer RFS compared with those with a high expression (n=26) (relapse rates of 46.2% and 7.7%, respectively; figure 2G; top; log-rank p=0.00113). The HPS was also significantly prognostic in the validation cohort using both univariate (HR 0.123, 95% CI (0.027 to 0.550), p=0.006; table 1) and multivariate analyses (HR 0.093, 95% CI (0.019 to 0.466), p=0.004; table 1), which equates to a >10-fold higher risk of relapse in the HPS-low group compared with the HPS-high. We confirm the subtype-specific nature of the HPS, as it again provides no clinical value in stratifying the LoFi population based on outcome (figure 2G; bottom; log-rank p=0.46596).

This validation cohort contained additional molecular features that were not available in our discovery cohort; however, we found no significant associations between the HPS and mismatch repair, CIMP or CIN status, nor mutations in *TP53*, *KRAS* and *BRAF* (online supplemental figure 4F). While the vast majority of HiFi tumours were CMS4, we found that there was also no significant difference in the proportions of the various CMS^3^ and colorectal intrinsic subtypes^27^ groups between HPS groups (online supplemental figure 4F), suggesting that our approach has identified HiFi-specific biology not identifiable using established genetic and transcriptional subtype analysis.

### STAT1-mediated biology defines relapse in HiFi tumours

**Figure 3:**
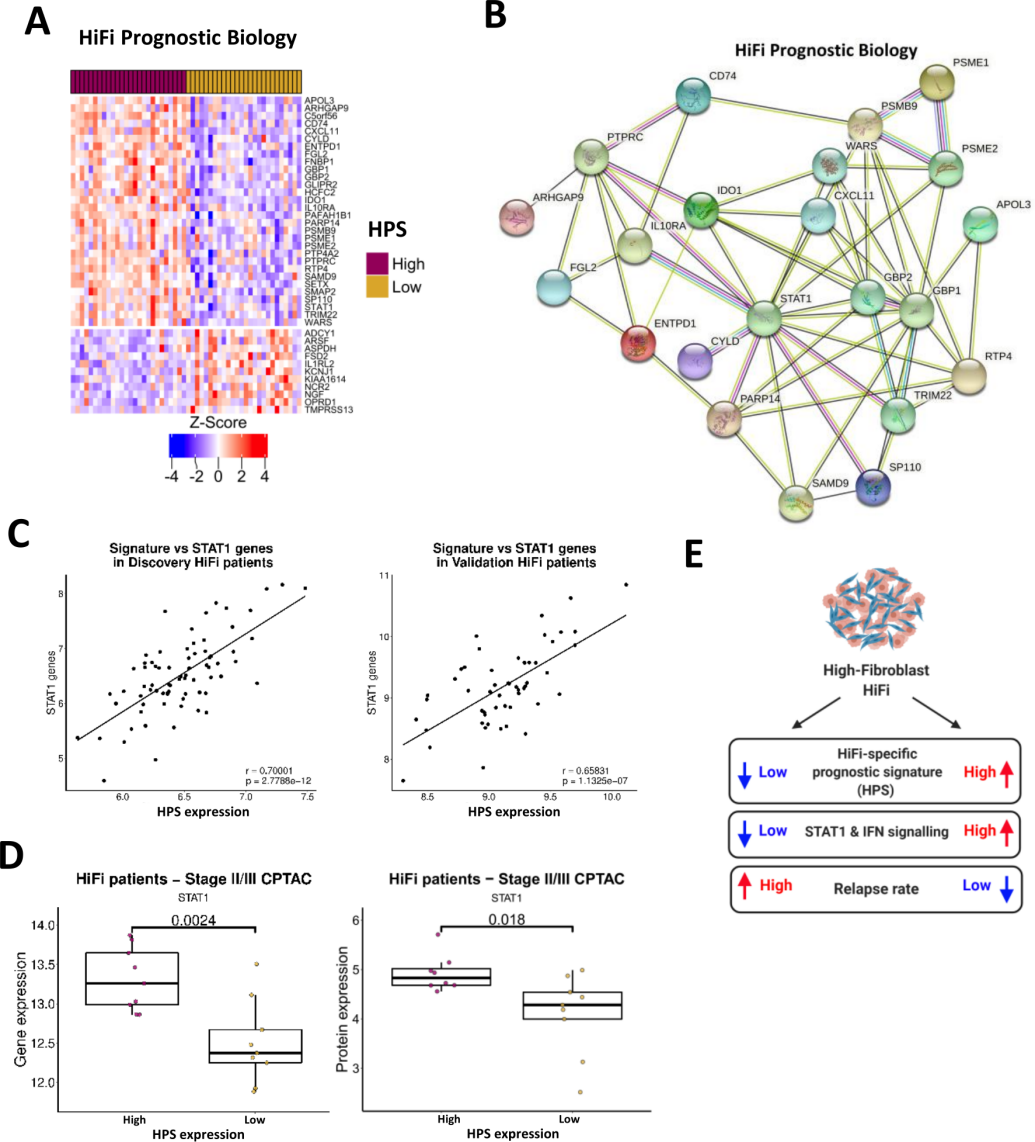
Validation of HiFi-specific prognostic biology and association with STAT1 (A) Heatmap displaying upregulated and downregulated genes shared by the differential comparisons between HPS expression groups in the discovery and validation cohorts (n=26 in each subgroup; 30 upregulated and 11 downregulated genes (adjusted p<0.05; table 2)). (B) String network formed by the upregulated genes form a cluster around STAT1. (C) Cumulative gene expression of *STAT1* and three of its target genes (*PSMB9, IRF1* and *TAP1*) correlated with expression of the HPS in the discovery (left) and validation cohort (right; t-test both p<0.00001). (D) Boxplots of *STAT1* gene expression (left) and protein levels (right) in HiFi patients in the CPTAC cohort according to HPS groups (high n=9 and low n=9; t-tests both p<0.05). (E) Schematic depicting the STAT1, IFN and relapse characteristics associated with the HiFi-specific prognostic signature within HiFi tumours. CPTAC, Clinical Proteomic Tumour Analysis Consortium; HiFi, high-fibroblast; HPS, HiFi-specific prognostic signature.

While our HPS was sufficient to stratify tumours into two groups based on RFS, we next investigated the overall differential biology underpinning HPS-high vs HPS-low tumours. Independent differential gene expression analyses were performed, revealing 41 genes significantly (BH adjusted p<0.05) differentially expressed in both discovery and validation cohorts; 30 upregulated and 11 downregulated genes in tumours with high signature expression (figure 3A, table 2).

**Table 2:** Differential biology identified by HiFi-specific prognostic signature

Upregulated genes	Discovery Fold change	Discovery Adjusted p value	Validation Fold change	Validation Adjusted p value
APOL3	1.479163484	0.038046826	1.530912747	0.019648112
ARHGAP9	1.967295618	0.028903696	1.592821689	0.023805238
C5orf56	1.32317962	0.042212342	1.400667094	0.005369033
CD74	1.32309124	0.046116242	1.576548039	0.027523104
CXCL11	1.92974677	0.034123781	5.00897748	0.008189299
CYLD	1.438201775	0.028903696	1.469132166	0.010754914
ENTPD1	1.794582609	0.024260819	1.376059763	0.020745822
FGL2	1.345513	0.021933613	2.600331326	0.003587353
FNBP1	1.469824152	0.027721291	1.71533174	0.009536017
GBP1	1.65880111	0.024260819	1.618328245	0.046475584
GBP2	1.647099662	0.024260819	1.781673902	0.002288833
GLIPR2	1.476671309	0.024260819	1.611068902	0.007753646
HCFC2	1.525739539	0.017725045	1.421761963	0.012742342
IDO1	1.73588083	0.048494417	2.994630603	0.000754221
IL10RA	2.055572033	0.017725045	1.654583304	0.019098469
PAFAH1B1	1.309143492	0.038004231	1.448782114	0.015670524
PARP14	2.105452644	0.03660005	1.524254089	0.036762555
PSMB9	1.270092042	0.046116242	1.666844663	0.036265881
PSME1	1.642023214	0.000168244	1.250842547	0.03164453
PSME2	1.603403275	0.024059724	1.314548729	0.039143267
PTP4A2	1.346153934	0.043074478	1.213154499	0.02914658
PTPRC	1.476678757	0.036567059	2.33082532	0.008189299
RTP4	1.485506409	0.024260819	2.15318341	0.000552526
SAMD9	1.688418114	0.043449318	1.977749961	0.036984171
SETX	1.750386764	0.022838328	1.487177653	0.006733321
SMAP2	2.686823125	0.03156768	1.403024067	0.036891535
SP110	2.000918379	0.000168244	1.455018839	0.012537887
STAT1	1.859897307	0.013017895	1.518148697	0.020017274
TRIM22	1.539257527	0.036567059	1.730531226	0.023710413
WARS	1.460735784	0.024260819	1.696493454	0.013432331

Downregulated genes	Discovery Fold change	Discovery Adjusted p value	Validation Fold change	Validation Adjusted p value
ADCY1	−1.21661236	0.03714883	−1.242515217	0.040619631
ARSF	−1.259331053	0.030430316	−1.205077753	0.044850161
ASPDH	−1.209403567	0.040310191	−1.224072938	0.047792544
FSD2	−1.205195523	0.038100021	−1.128329641	0.04727427
IL1RL2	−1.181897912	0.047573384	−1.295819216	0.031084084
KCNJ1	−1.171753534	0.038739777	−1.121058095	0.029132685
KIAA1614	−1.239454355	0.031957568	−1.198516417	0.021006687
NCR2	−1.223150807	0.040766339	−1.16135162	0.046766842
NGF	−1.234256437	0.033460361	−1.283709027	0.006961439
OPRD1	−1.127349469	0.043716659	−1.188396135	0.044656705
TMPRSS13	−1.268170072	0.025078493	−1.34200588	0.0429152

HiFI, high-fibroblast.

Using the STRING database (string-db.org) to identify and visualise interactions, upregulated genes formed a network around STAT1 (figure 3B). As these analyses are based on total gene expression levels for *STAT1* itself; to assess downstream activation, we next examined *STAT1* in combination with several of its target genes (*STAT1, PSMB9* (*LMP2*), *IRF1* and *TAP1)*, where we observed strong direct positive correlation between their expression and the HPS in both discovery and validation cohorts (figure 3C; Pearson’s Correlation r=0.70001 and r=0.65831, (online supplemental figure 4G). Furthermore, stratification of an additional independent cohort of stage II/III colon patients (Clinical Proteomic Tumour Analysis Consortium, CPTAC)^28^ into HiFi and LoFi using our fibroblast score, followed by sub-stratification using the HPS, validated a significant enrichment for total STAT1 gene and protein expression in HiFi patients with high HPS expression (figure 3D; t-test p=0.0024 and p=0.018). Although these signalling pathways can be an indication of general tumour infiltration levels, we demonstrated that patient stratification based on the ESTIMATE ImmuneScore^17^ is insufficient for prognostic stratification when applied specifically to HiFi patients, in either the discovery or validation cohorts (online supplemental figure 5A); left), and does not consistently align to HPS (online supplemental figure 5A; right). Furthermore, comparisons of the relative abundance of immune cells in the discovery and validation cohorts, using the CIBERSORT tool,^29^ revealed a significantly larger proportion of dendritic cells (DCs) that was only apparent in the discovery cohort HPS-high patients versus HPS-low and not recapitulated in the validation cohort (online supplemental figure 5B); t-test p=0.00076 and p=0.51333).

In summary, our HiFi-specific analyses identified that elevated expression of HPS, which distinguished primary tumours based on IFN-alpha, IFN-gamma and STAT1-related biological signalling, was significantly associated with disease relapse specifically within stroma-rich CC (figure 3E).

### HiFi specific STAT1-related prognostic biology is associated with higher levels of immune lineage-specific antigen processing and presentation

**Figure 4:**
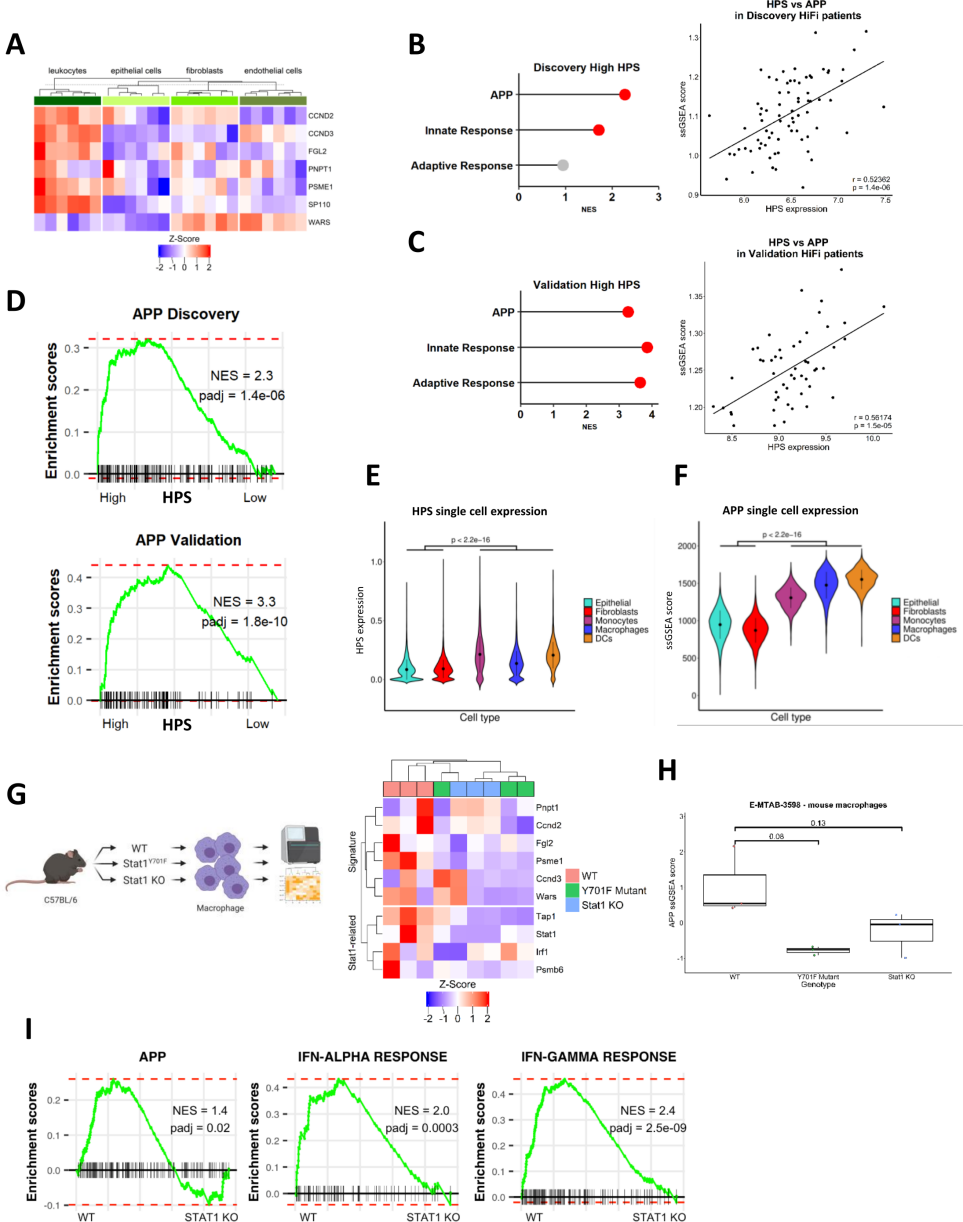
CRC tumour single-cell data confirms immune-specific nature of signature. (A) Gene expression of individual genes within the HPS according to a public dataset of CRC cell lineages purified by fluorescence-activated cell sorting (FACS) (n=4 populations from n=6 patients; total n=24). (B) Enrichment for APP, adaptive and innate signalling in HPS high group compared with low in HiFi tumours from the discovery cohort (left) (red=adjusted p<0.05). Correlation between ssGSEA scores for APP and HPS gene expression in the discovery cohort (Pearson’s correlation r=0.5, p=1.4e-06; right). (C) Enrichment for APP, adaptive and innate signalling in HPS high group compared with low in HiFi tumours from the validation cohort (left). Correlation between ssGSEA scores for APP and HPS gene expression in the validation cohort (Pearson’s correlation r=0.6, p=1.5e-05; right). (D) Enrichment for APP using pairwise GSEA in HPS high group compared with low in HIFI tumours from both the discovery and validation cohorts. (E, F) Immune cell populations have significantly higher expression of the HPS (E) and GO APP ssGSEA scores (F) than epithelial cells and fibroblasts (t-test both p<2.2e-16). (G) Expression levels of HPS genes and STAT1-related targets and (H) APP ssGSEA scores in bone-marrow derived macrophages with either wild-type (WT), mutant (Y701F mut) or knockout (KO) *STAT1* (n=3 for each genotype) (t-test). (I) Pairwise GSEA for GO APP, interferon alpha and gamma response in WT V STAT1 KO mouse macrophages. (n=3 per genotype). APP, antigen processing and presentation; CRC, colorectal cancer; GSEA, gene set enrichment analysis; HiFi, high-fibroblast; HPS, HiFi-specific prognostic signature; ssGSEA, single-sample GSEA.

Using transcriptional data derived from leucocyte, epithelial, fibroblast and endothelial lineages isolated from colorectal tumour tissue (GSE39396),^30^ we determined that six of the seven HPS genes were highly associated with tumour infiltrating immune lineages compared with the other cell types (figure 4A). In line with functionally active STAT1 signalling (figure 3C), we observed increased expression of major histocompatibility (MHC) class I receptors, *HLA-A*, *HLA-B* and *HLA-C*, associated with HPS in both cohorts (online supplemental figure 5C); t-test p<0.05, above and below median HPS; *HLA-B* was not present on array used in the discovery cohort). In addition, elevated adaptive and innate immune signalling, alongside ssGSEA gene ontology scores for the antigen processing and presentation (APP) machinery (figure 4B–D, (online supplemental figure 5D) were all associated with high HPS. We next examined the association between HPS expression and APP specifically within purified immune lineages (GSE24759),^31^ which revealed a significant and strong positive correlation between HPS expression (originally identified from bulk tumours; figure 4B–C) and APP signalling in mature antigen presenting cells (APC) (online supplemental figure 5E; Pearson’s correlation r=0.89974, p=1.36×10^−11^). Interrogation of single-cell RNA-Seq data from tumour-infiltrating immune populations isolated from a further independent cohort of CRC tumours,^32^ which again confirmed a significant elevation of HPS expression (figure 4E; t-test p<0.0001) and APP signalling (figure 4F; t-test all p<0.0001) in tumour infiltrating monocytes, macrophages and to a greater extent in DCs compared with epithelial and CAF populations.

Using transcriptional data derived from bone marrow-derived macrophages (BMDM) isolated from WT, *Stat1*Y701F (dominantnegative) or *Stat1*-/- mice (E-MTAB-3598),^33^ we confirmed the essentiality of functional STAT1 in regulating gene expression of HPS and STAT1 targets (figure 4G), APP signalling using ssGSEA (figure 4H; t-test) and APP, IFN-alpha and IFN-gamma response signalling using pair-wise GSEA (figure 4I).

### HPS signalling is associated with double stranded RNA and viral response cascades

**Figure 5:**
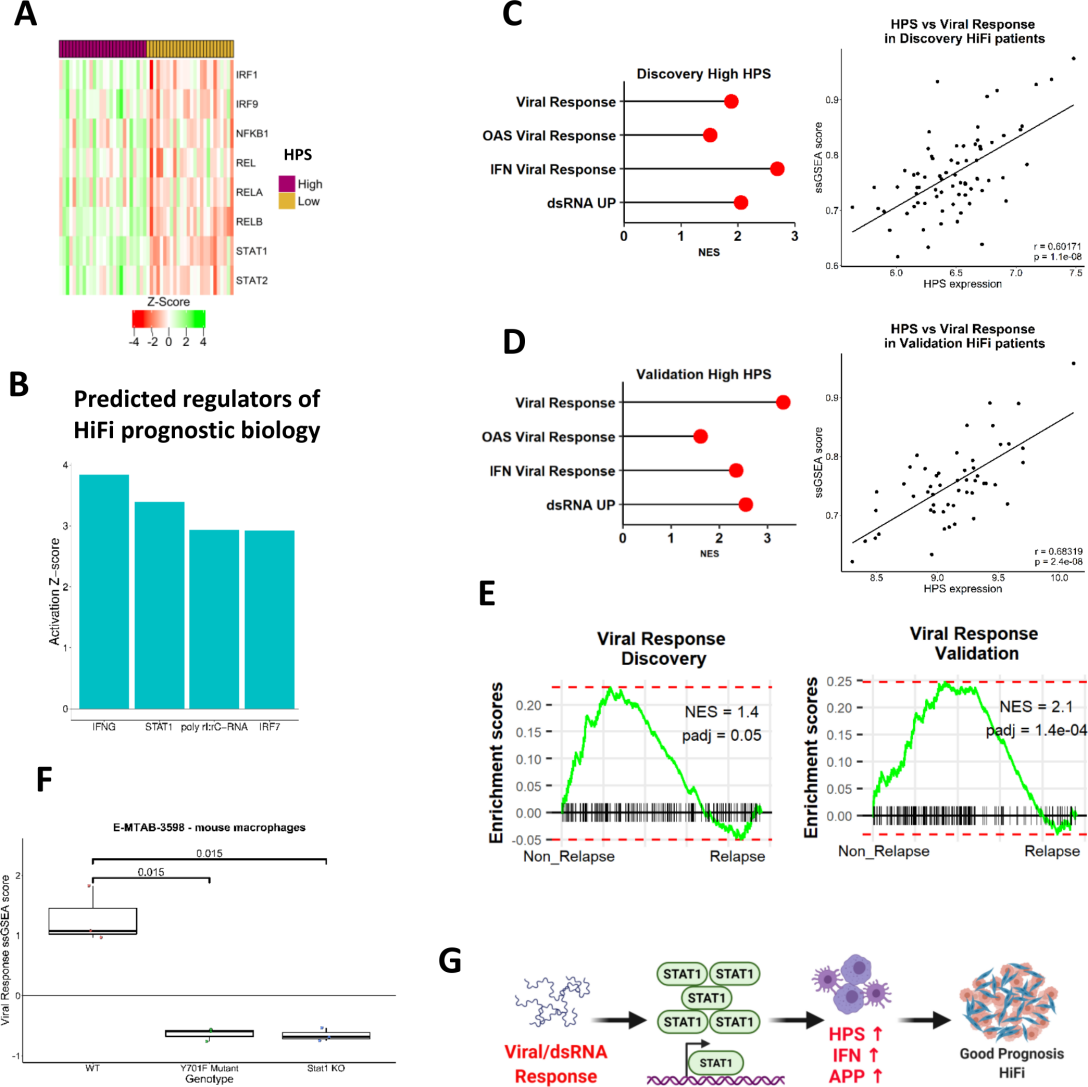
IFN and APP signalling cascades are associated with a STAT1-mediated viral/dsRNA response. (A) Activity status of key TF regulons according to HPS groups in the validation cohort (n=26 in each subgroup). (B) Top upstream regulators from an ingenuity pathway analysis (IPA) of the HPS differentially expressed genes in both the discovery and validation cohorts (table 2). (C) Enrichment for multiple viral response gene sets and dsRNA response in HPS high group compared with low in HiFi tumours in the discovery cohort (red=adjusted p<0.05; left). Correlation between ssGSEA scores for viral response and HPS gene expression in the discovery cohort (Pearson’s correlation r=0.6, p=1.1e-08; right). (D) Enrichment for multiple viral response gene sets and dsRNA response in HPS high group compared with low in HiFi tumours in the validation cohort (left) (red=adjusted p<0.05). Correlation between ssGSEA scores for viral response and HPS gene expression in the validation cohort (Pearson’s correlation r=0.7, p=2.4e-08; right). (E) Enrichment for viral response using pair-wise GSEA in non-relapse versus relapse HiFi tumours from both the discovery and validation cohorts. (F) viral response ssGSEA scores in bone-marrow derived macrophages with either wild-type (WT), mutant (Y701F mut) or knockout (KO) *STAT1*. (n=3 for each genotype) (t-test p<0.05). (G) Schematic detailing role for viral response/dsRNA signalling in regulating STAT1-mediated signalling cascades, HPS, APP and IFN signalling in immune lineages results in a good prognosis HiFi tumour. APP, antigen processing and presentation; CRC, colorectal cancer; ds RNA, double stranded RNA; HiFi, high-fibroblast; HPS, HiFi-specific prognostic signature; ssGSEA, single-sample GSEA; TF, transcription factor.

Transcription factor (TF) activity prediction, using the DoRothEA resource, to identify potential regulons responsible for the signalling and phenotypes associated with HPS in HiFi tumours (online supplemental figure 6A) revealed a strong association with *STAT1, STAT2*, IFN (*IRF1, IRF9*) and NFκB (*NFKB1, REL, RELA, RELB*) TFs (figure 5A). In parallel, we used ingenuity pathway analysis in conjunction with the HPS differential genes identified earlier (figure 3A) to predict upstream regulators of the HiFi-specific prognostic biology, and in line with our findings thus far, interferon gamma (*IFNG*), *IRF7* and *STAT1* were all identified (figure 5B). In addition, the synthetic double stranded RNA (dsRNA) viral mimetic and TLR3 agonist, Poly(I:C), was also identified as an upstream regulator of, and potential therapeutic agent to activate, the STAT1-mediated signalling and APP phenotypes associated with prognosis in HiFi tumours (figure 5B). Poly(I:C) is a potent immune adjuvant via viral-mimicry that can be safely used for inducing both a transient innate immune response and maintained adaptive response, which notably is the same signalling we found was associated with the HPS (figures 4–5). We next investigated upstream events that could trigger the differential STAT1-mediated innate/adaptive immune activity and APP, and in line with poly(I:C) findings, these analyses revealed an enrichment of signalling associated with a viral response and the presence of dsRNA in non-relapsing HiFi tumours, with high HPS expression (figure 5C–E). Furthermore, this viral response relies on the presence of functional STAT1, emphasising the importance of this signalling cascade (figure 5F; t-test p<0.05).

Taken together, these data confirm the biology underpinning the bulk tumour-derived HPS is significantly associated with functional STAT1 activity and APP in tumour-infiltrating professional APC in CC, which may be downstream of a dsRNA and/or viral response in a subset of HiFi tumours (figure 5G).

### The TLR3 agonist poly(I:C) elevates mechanistic phenotypes associated with improved outcome in HiFi CRC

**Figure 6:**
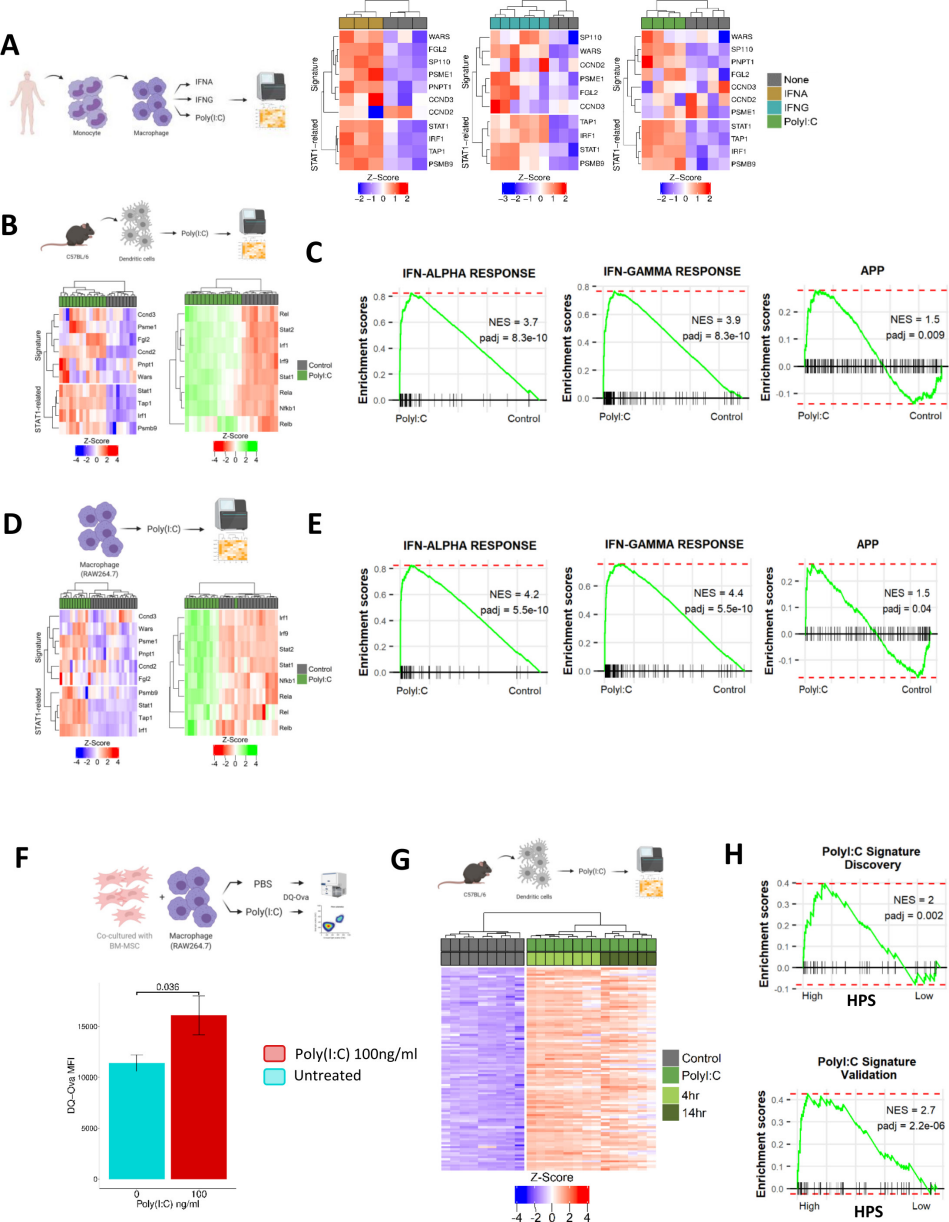
The TLR3 agonist poly(I:C) could be a potential treatment for HiFi (A) gene expression of HPS and STAT1 targets in human macrophages from different datasets treated with interferon (IFN) alpha (left) (n=3), IFN gamma (middle) (n=6) and poly(I:C) (right) (n=4) compared with untreated control samples (n=>3). (B) gene expression of HPS and STAT1 targets (left) and TF activity (right) in dendritic cells from mice treated with poly(I:C) (n=14) or untreated. (C) pair-wise GSEA of IFN alpha and gamma response, alongside APP gene sets in dendritic cells from mice treated with poly(I:C) or untreated. (D) Gene expression of HPS and STAT1 targets (left) and TF activity (right) in raw macrophage cells treated with poly(I:C) (n=12) or untreated. (E) Pair-wise GSEA of IFN alpha and gamma response, alongside APP gene sets in RAW macrophage cells treated with poly(I:C) or untreated. (F) Flow cytometry analysis of antigen processing in a co-culture comprised of primary mouse mesenchymal stromal cells (MSCs) and the mouse macrophage cell line RAW264.7, incubated with fluorescently labelled ovalbumin protein (DQ-Ova) and treated with either poly(I:C) or control (n=3) (t-test p<0.05). (G). differentially expressed genes (logFC >2 and adjusted p<0.001) in Poly(I:C) treated vs non-treated dendritic cells creating the ‘Poly(I:C) Signature’. (H) Enrichment for Poly(I:C) Signature using pair-wise GSEA in HPS high group compared with low in HiFi tumours from both the discovery and validation cohorts. APP, antigen processing and presentation; HiFi, high-fibroblast; HPS, HiFi-specific prognostic signature; TF, transcription factor.

Testing of IFN-alpha (IFNA), IFN-gamma (IFNG) or poly(I:C) in primary human macrophage immune lineages (GSE46599, GSE1925 and GSE41295) confirmed their ability to induce expression of the HPS genes, alongside increased expression of *STAT1* and its target genes (figure 6A). A therapeutic form of poly(I:C) has recently demonstrated favourable safety characteristics in a number of phase I clinical trials^34 35^; therefore, we selected poly(I:C) for further testing. Using a mouse DC model (GSE46478), we observed increased expression of the HPS genes and STAT1-related genes on treatment with poly(I:C), alongside significant induction of the same STAT, IFN and NFκB regulons (figure 6B) and IFN-alpha response, IFN-gamma response and APP associated with prognosis in HiFi tumours (figure 6C). These results were further confirmed using the RAW264.7 macrophage model (GSE15066; figure 6D and E).

Furthermore, to complement this transcriptional signalling, and to validate the utility of the *in silico* measure of APP, we next performed *in vitro* phenotypic measurements of antigen processing, using a fluorescent-labelled ova protein (DQ-ova) in the RAW264.7 macrophage model, cocultured with tumour-conditioned primary mesenchymal stromal cells to represent the stromal environment of a HiFi tumour microenvironment (TME) (figure 6F). In support of the potential therapeutic relevance of poly(I:C) in this setting, macrophages from the poly(I:C) treated cocultures had significantly higher DQ-ova fluorescence, and therefore induced antigen processing, in this model (figure 6F; t-test p=0.036, (online supplemental figure 6BC). To assess if the key characteristics associated with HPS in bulk tumour samples can be induced following a dsRNA/Poly(I:C) response in immune lineages, we created a ‘Poly(I:C) Signature’ of n=75 (human) differentially expressed genes from the Poly(I:C) treated DCs (figure 6B) (logFC >2 and adjusted p<0.001) (figure 6G, (online supplemental table 4). Using GSEA according to HPS subgroups in HiFi samples, the Poly(I:C) signature was significantly enriched in both the discovery and validation cohorts in HPS-high compared with HPS-low (figure 6H, (online supplemental figure 6D), further confirming that the biology underpinning HPS can be therapeutically induced via a viral-like dsRNA-response in immune cells.

**Figure 7:**
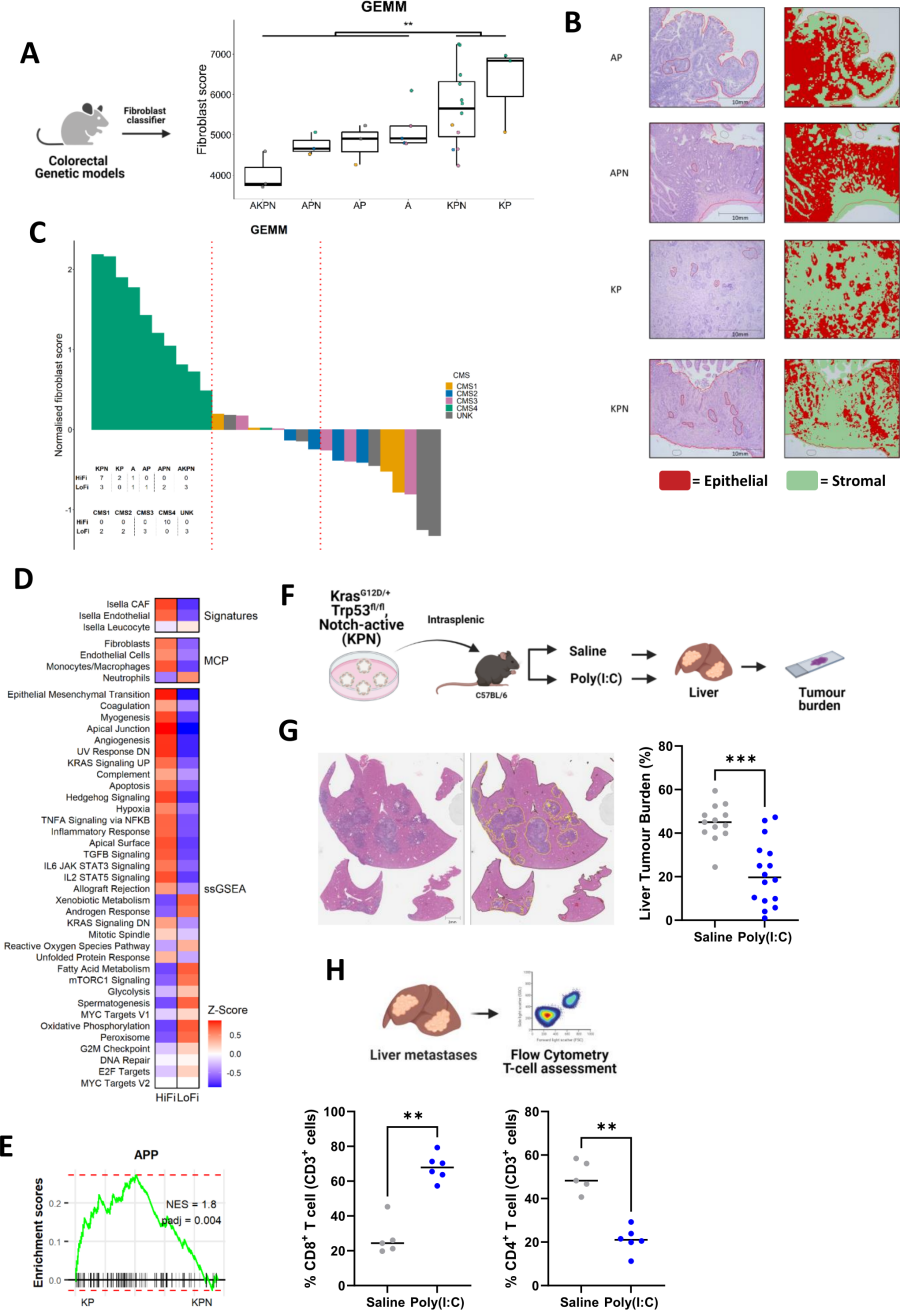
In vivo validation of poly(I:C) in HiFi model (A) CMS classification according to fibroblast score of GEMM genotypes. (A=*Apc* ^fl/fl^, K=*Kras* ^G12D/+^, p=*p53* ^fl/fl^ and n=*Notch1Tg*/^+^) (t-test p<0.01). (B) Stromal scores from H&E slides using digital pathology applied to GEMM tissue. (C) Waterfall plot of fibroblast scores indicating CMS classification in GEMMs. (D) comparison of HiFi (n=10) and LoFi (n=10) GEMMs using previously published stromal signatures and gene sets as assessed in figure 1G. (E) Pairwise GSEA of the APP gene set in CMS4 KP compared with CMS4 KPN. (F) Intrasplenic metastasis assay with KPN models in vivo treated with poly(I:C) compared with saline control. (G) Digital pathology assessment of H&E from in vivo studies demonstrates reduced liver metastasis in mice treated with poly(I:C) (n=16) compared with saline control (n=13) (Mann-Whitney U test). (H) Flow cytometry assessment of CD3 +cell populations from liver metastases in treatment groups highlight significant elevation of CD8 +T cells alongside significant reduction in CD4 +T cells in poly(I:C) arm (n=6) compared with saline (n=5) (Mann-Whitney U test; both p<0.05). APP, antigen processing and presentation; CMS, consensus molecular subtypes; GEMM, genetically engineered mouse model; HiFi, high fibroblast; LoFi, low fibroblast. *** denotes p<0.0002, ** denotes p<0.05.

While previous studies have described the efficacy of poly(I:C) in tumour models, predominantly melanoma, its ability to reduce metastases in a CMS4-related genetically engineered mouse model (GEMM) has not been tested. To this end, we assessed a range of previously characterised GEMMs to identify genotypes associated with HiFi transcriptional signalling and histology. These analyses revealed that the recently developed stroma-rich CMS4 models; *Kras^G12D/+^*, *Trp53^fl/fl^* (KP) and KP with constitutively activated NOTCH1 intracellular domain (KPN)^36^ display significantly higher fibroblast scores (figure 7A) and stromal histology (figure 7B) compared with a number of *Apc*-based models.

In line with our discovery and validation human cohorts (figure 1E and online supplemental figure 4C), we saw a strong association between CMS4 classification and fibroblast scores (figure 7C). In addition, assessment of the same signals observed in HiFi versus LoFi human tumours (figure 1G, (online supplemental figure 4E) revealed an analogous pattern of enrichment in HiFi-related signalling cascades such as EMT, myogenesis and TGF-β signalling in HiFi/CMS4 GEMMs when compared with LoFi GEMMs (figure 7D). While both KP and KPN models were associated with HiFi/CMS4 classification, the KPN model was most representative of a poor prognostic HiFi model given its previously-reported highly metastatic nature.^36^

In line with this poor prognosis, we observe a significantly reduced APP signalling in CMS4 KPNs compared with the CMS4 KP models (figure 7E; NES=1.8). We, therefore, selected the KPN model to test the in vivo efficacy of poly(I:C) in reducing metastatic tumour burden using an intra-splenic injection metastatic assay (figure 7F). Following splenic KPN implantation, treatment with poly(I:C) (4 mg/kg administered biweekly by intraperitoneal injection from 9 to 42 days post-surgery) significantly reduced liver metastases burden in vivo, as assessed using a digital histology assessment (figure 7G; pooled in vivo results Mann-Whitney U p<0.0002). (online supplemental figure 7A); individual in vivo experiments), validating our in silico and in vitro analyses, alongside supporting its clinical translation in this setting. At endpoint, FLOW analyses of liver metastases (online supplemental figure 7B) revealed a significant elevation of CD3 +CD8+cytotoxic T cells and a complementary significant reduction in CD3 +CD4+T cells in poly(I:C)-treated mice compared with saline control (figure 7H; Mann-Whitney U both p<0.05).

## Discussion

**Figure 8:**
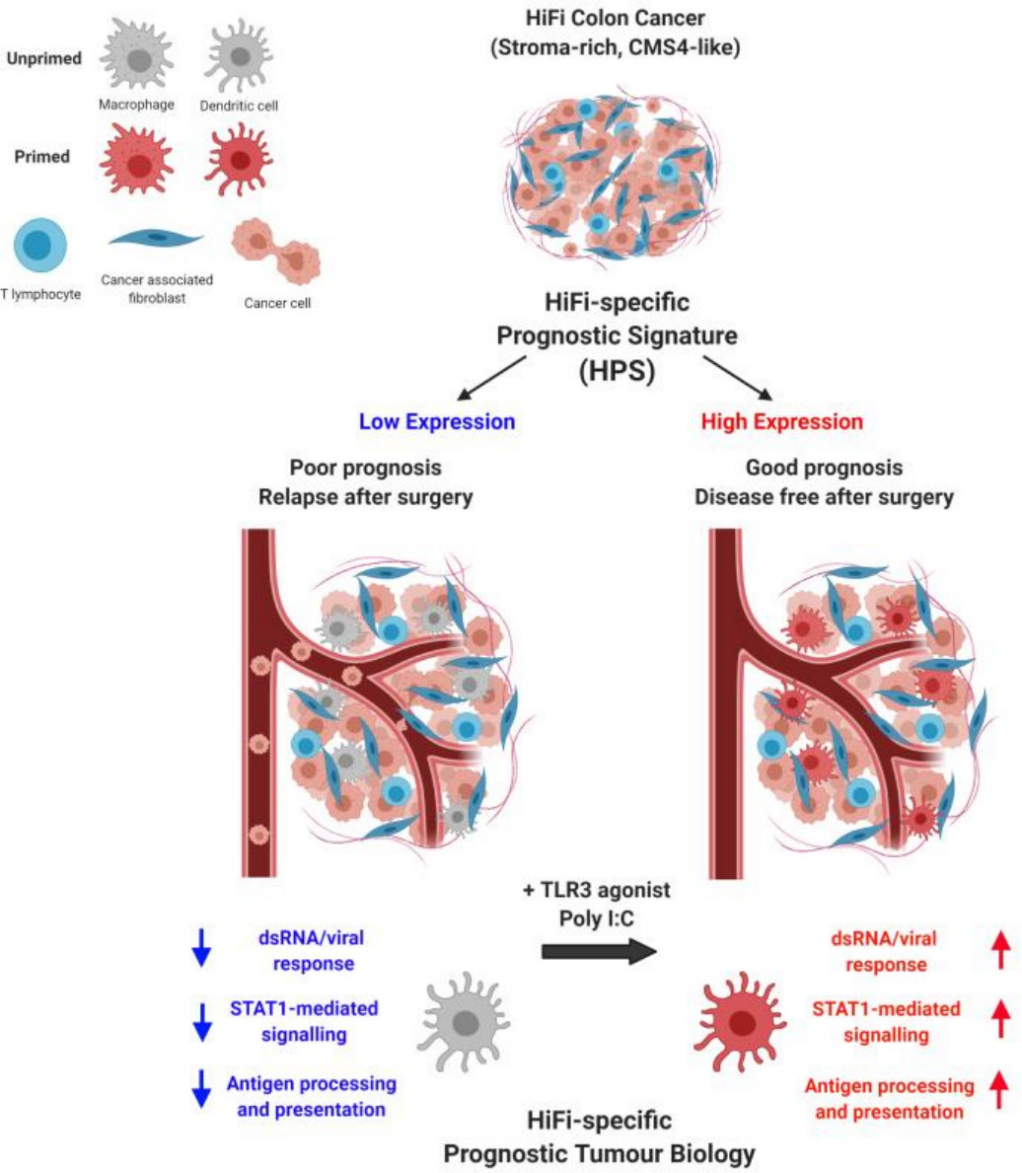
Graphical summary

We and others have previously demonstrated how CRC subtypes are heavily influenced by the composition of the TME, and the significant association with poor prognosis in the fibroblast-rich, CMS4 and stem-like subtype.^37–42^ When compared with epithelial-rich subtypes, stroma-rich tumours display elevated signalling related to TGF-β and other stromal biologies, and this elevated signalling in general has been used as the rationale for targeting these pathways as potential therapeutic options. While substantial preclinical data supports TGF-β blockade as a promising target, the positive results obtained in *in vitro* and *in vivo* studies have not translated into clinical efficacy for stroma-rich tumours, even after numerous clinical trials in the past decade.^43 44^ Currently there are significant efforts aimed at combining TGF-β blockade with immunotherapy, which may yet yield clinical benefit. However, in this study we reveal that the biology associated with disease relapse *within* stroma-rich tumours (and which are uniformly elevated for TGF-β signalling) is *not* associated with the biology that distinguishes between stroma-rich and epithelial-rich subtypes, nor is it associated with factors that are prognostic in general across unstratified stage II/III CC cohorts. Therefore, we set out to identify prognostic biology underpinning relapse specifically *within* stroma-rich tumours, and to use this new understanding to identify therapeutic vulnerabilities that could be exploited to reduce relapse rates in this poor prognostic patient group. This approach revealed that elevated levels of STAT1-mediated APP signalling downstream of a viral/dsRNA response in immune lineages correlated with improved RFS only in HiFi tumours; signalling that provides no prognostic value in the relatively good prognostic LoFi group. Furthermore, we demonstrate the therapeutic potential of this biology, as treatment with poly(I:C) resulted in elevation of the signalling and phenotype associated with good prognosis in HiFi tumours and, most importantly, a significant reduction of liver metastases in a mouse model of stroma-rich CC. Data presented here reveals a subtype-specific therapeutic approach, mediated via poly(I:C), that could potentially improve outcome for patients in the poor prognostic, high-fibroblast subtype of early stage CC (figure 8).

Ontology/pathway-led approaches for transcriptional analyses have the advantage of identifying biologically meaningful information associated with a particular subgroup, in our case relapse within HiFi tumours, rather than individual genes which can be confounded by issues such as intratumoural heterogeneity^39 45^ or technical variations between profiling platforms/methods.^46^ In addition, our study was designed to identify elevated phenotypes, and their regulators, associated with improved outcome to inform new treatment options that boost this biology; an approach that is known to deliver increased biological insights and more successful therapeutic outcomes when compared with those that rely on trying to downregulate or repress biology, which can be confounded by off-target effects.^47^ The holistic discovery approach used here, which incorporates biological knowledge using experimentally validated signatures from the Hallmarks collection,^48^ indicated that STAT1-mediated APP downstream of viral/dsRNA response was associated with reduced relapse rates in HiFi tumours, which in turn could be induced through treatment with IFNA, IFNG and the dsRNA TLR3 agonist, poly(I:C).

Interferon therapies have been trialled in multiple cancer types, but efficacy has been hindered by dose-limiting toxicities.^49^ Trials using TLR agonists can induce IFN production, while causing fewer side effects compared with exogenous IFN treatment.^49^ Following treatment with poly(I:C), a number of human or mouse macrophages and DCs display the same transcriptional signalling and APP activation that distinguished good from poor prognostic HiFi tumours and was sufficient to reduce metastatic lesions in a HiFi-specific mouse model. A recent breast cancer study also demonstrated that the prognostic value of DC subsets was dependent on the subtype of the tumours themselves, as signatures specific to plasmacytoid DCs and cDC2 cells were prognostic in triple-negative breast cancers, but not in the luminal subtype.^50^ In line with this, the biology we identify as associated with prognosis in HiFi tumours provides no prognostic value in LoFi patients.

Elevation of dsRNA and viral response signalling was observed in HiFi tumours with reduced relapse rates and may provide a biological explanation for the differential activation status of this STAT1 and IFN-related biology in the different subsets of HiFi tumours. Activation of these same cascades were noted in a recent pancreatic cancer study as a downstream consequence of increased expression of endogenous retroviral transcripts.^51^ In our study, we highlighted the essential nature of functional STAT1 in regulating this potential viral mimicry signalling in specific immune lineages and in the APP phenotype associated with improved outcome in HiFi tumours. Increased abundance and functionality of tumour infiltrating DCs correlate with prognosis in a variety of cancers,^52^ and while dsRNA and viral signalling can drive their activation, we cannot rule out that these DCs are also regulated by other undiscovered TME-related factors within our study, including cytokines and other soluble factors. Data presented here have revealed a number of critical biological cascades underpinning poor prognosis in HiFi tumours, however additional secretome, epigenetic and microbiome profiling would be of interest to identify factors that regulate activation and survival in specific immune lineages, or in the case of DCs, by quantification of factors regulating their commitment, turnover and dendropoiesis.

Expansion and activation of DC lineages, via pretreatment with the growth factor FLT3L, which promotes DC commitment in haematopoietic progenitor cells and subsequent DC activation and growth, followed by poly(I:C) treatment, has demonstrated utility as an approach to improve response to checkpoint blockade in melanoma models.^53^ Very recent data on the safety and efficacy of neoadjuvant immuno-oncology treatment (combined CTLA4 and PD1 blockade) in CC confirm the shifting clinical landscape for neoadjuvant treatment scheduling for localised colon tumours.^54^ Results from that clinical trial indicate that while microsatellite instability-high tumours universally displayed pathological response to treatment, only 27% of microsatellite stable tumours were responsive; a group that urgently requires therapeutic interventions that can reprime the suppressed innate immune system and ultimately reinitiate tumour immune surveillance. Taken together, our subtype-specific *in silico* data, alongside the *in vitro* and *in vivo* data presented here, strongly support the clinical testing of poly(I:C) as a novel treatment option in the neoadjuvant setting to complement the current adjuvant standard of care, to reduce relapse rates for patients with stroma-rich CC.

In conclusion, our tumour-based discovery and validations, alongside *in vitro* and *in vivo* models, have identified a key role for viral/dsRNA response and IFN signalling, upstream of a STAT1-mediated cascade, which in turn drives an innate-adaptive immune activation, as a critical mediator of relapse in stroma-rich CRC. Data presented here provide a strong biological rationale for clinical testing of poly(I:C) as a novel therapeutic option to reduce metastatic relapse rates in the worst prognostic group of early stage CC.

## Methods

Additional Methods details are available within online supplemental material 1. A study overview including methods and criteria used is presented in online supplemental figure 8. Schematics designed using BioRender.com.

10.1136/gutjnl-2021-326183.supp3 Supplementary data

### Patient datasets and data processing

The discovery transcriptional dataset was previously assembled for the development of the FDA-approved stage II ColDx/GeneFx assay,^20^ consisting of 215 stage II CC patients (ArrayExpress accession number E-MTAB-863). As this was an existing cohort, we did not perform sample size power calculations. Clinicopathological information is in online supplemental table 2). The stage II/III untreated CC validation dataset (GSE39582) was downloaded as CEL files from GEO, processed then collapsed in the same way as the discovery dataset (detailed further in online supplemental methods). Clinicopathological information is in online supplemental table 3. RNA-seq and label-free proteomic data from the CPTAC^28^ colon adenocarcinoma cohort (n=100) were downloaded from http://linkedomicsorg/cptac-colon/. The use of patient material from the S:CORT programme was approved by the ethics commission (REC 15/EE/0241). GSE39396: Fluorescence-activated cell sorted (FACS) purified cells (CD45 +leukocytes, FAP +fibroblasts, CD31 +endothelial cells and Epcam +epithelial cells) from 6 CRC patients. Data were retrieved from GEO in its log2 RMA normalised form.

### Survival analysis

All survival analyses were performed in R using the survival package (V.3.2–13). For Kaplan-Meier curves and Cox proportional hazards models, median value of gene expression was used to dichotomise patients into high/low groups. In the discovery cohort, univariate Cox proportional hazards regression analysis was performed to identify genes that correlated with RFS. Genes with a likelihood p<0.20 were subjected to multivariate Cox analysis adjusted for age, sex, pT stage, tumour location, tumour differentiation grade, tumour subtype (mucinous/non-mucinous), lymphovascular invasion and the number of lymph nodes. A total of 214 samples were included (one sample was removed due to lack of clinicpathology information). The multivariate analysis in the validation dataset was adjusted for age, sex, TNM stage and tumour location. All the variables in the model were non-significant (p>0.05) and proportional hazards assumptions were met for both the discovery and validation cohorts using cox.zph function.

### Immune lineage datasets

GSE24759: 38 purified populations of human haematopoietic cells.^31^ GSE46599: primary human monocytes differentiated into macrophages and treated with interferon alpha.^55^ GSE1925: primary human monocytes which were differentiated into macrophages and treated with interferon gamma.^56^ GSE41295; primary human monocytes differentiated into macrophages and treated with poly(I:C).^57^ GSE46478: primary DCs from C57BL/6 mice.^58^ GSE15066: mouse macrophage cell line RAW264.7 stimulated with poly(I:C).^59^ E-MTAB-3598: BMDM isolated from WT, Stat1^Y701F^ or Stat1^-/-^ mice.^33^ All of these datasets were collapsed to gene-level using the same method for patient data outlined in online supplemental methods section.

The generation of the single-cell RNA-Seq data has been previously published^32^ and is detailed in online supplemental methods.

### GEMM dataset descriptor and histology

Detailed information is available in online supplemental methods. Briefly, all animal experiments were performed in accordance with a UK Home Office Project Licence (70/9112), observed ARRIVE guidelines and were reviewed by local animal welfare and the ethical review committee at the University of Glasgow. Histology assessments were performed on a previously established and described cohort as part of the ACRCelerator programme (https://www.beatson.gla.ac.uk/ACRCelerate/acrcelerate.html).

### KPN intrasplenic model

Intrasplenic injection was performed as previously described (Jackstadt *et al*)^36^ using a cell suspension of liver metastasis organoids derived from a single C57BL/6 *Kras^G12D/+^*, *Trp53^fl/fl^*, constitutively activated NOTCH1 (KPN) mouse. Organoid donor and recipient mice were sex and strain matched. Nine days postimplantation mice were administered poly(I:C) at 4 mg/kg in saline by biweekly intraperitoneal injection (n=6) or saline vehicle control (n=6; n=5 used for tissue processing) until sampling on day 42. Blind to treatment, body weight and liver weight was recorded and gross liver metastasis quantified. The study was repeated with n=12 poly(I:C) and n=12 saline with similar results.

### Mouse model tissue processing

A biopsy of liver metastasis was taken for flow cytometry with the remainder fixed in 10% neutral buffered formalin and processed by standard histological processing into paraffin. 5 µm sections were cut and stained for H&E.

## Sample processing and staining for flow cytometry

A detailed protocol and gating strategy is included in the online supplemental methods, online supplemental figures section. Analysis was conducted on FlowJo V.10.7.2. Cells were gated based on live cell status from live/dead stain, single cells and CD45 +cells selected. From here, data was down-sampled to 12 000 events per sample and CD3 +CD4+ and CD3+CD8+ cells were identified.

## Metastasis scoring

H&E sections of liver were scanned using an Aperio AT2 slide scanner at 20 x and svs files imported) into QuPath v0.2.3. A pixel thresholder (Resolution: 4.02 µm/px; Channel: Average channels; Prefilter: Gaussian; Smoothing sigma: 3.0; Threshold: 200.0) was applied to quantify the total tissue area. Liver metastasis were manually annotated on each whole slide image. The mean tissue and liver metastasis area was utilised to calculate a tumour burden percentage per mouse.

10.1136/gutjnl-2021-326183.supp2 Supplementary data

10.1136/gutjnl-2021-326183.supp4 Supplementary data

## Data Availability

Data are available in a public, open access repository.
