# Supplementary material for: Activation of innate-adaptive immune machinery by poly(I:C) exposes a therapeutic vulnerability to prevent relapse in stroma-rich colon cancer
Source: Gut. 2022 Apr 27;71(12):2502–17. doi: 10.1136/gutjnl-2021-326183 (PMC9664095; doi:10.1136/gutjnl-2021-326183)
Supplement: Supplementary data [file gutjnl-2021-326183supp001.pdf]

Supplementary Figure 1

A

Cut1	HR1	P1
1473.97	0.909811877	0.812063248
1512.056241	0.669377513	0.219964264
1550.142482	0.750410578	0.380201791
1588.228723	0.791699836	0.475315669
1626.314964	0.833520265	0.577835972
1664.401205	0.875883881	0.685450566
1702.487446	0.875883881	0.685450566
1740.573687	0.822405819	0.522782527
1778.659928	0.871546038	0.643757589
1816.746169	0.83781441	0.541295664
1854.83241	0.906726625	0.729292178
1892.918651	1.037528078	0.896398444
1931.004892	1.09257795	0.749181528
1969.091133	1.103278018	0.712536404
2007.177374	1.166347115	0.564046108
2045.263615	1.13913282	0.619640478
2083.349856	1.056373612	0.829774604
2121.436097	1.121894773	0.648049994
2159.522338	1.136163904	0.608429835
2197.608579	1.282838944	0.317540457
2235.69482	1.326277252	0.252390041
2273.781061	1.422476785	0.153210865
2311.867302	1.594979363	0.056234447
2349.953543	1.567089383	0.062123833
2388.039784	1.521138426	0.077987899
2426.126025	1.666354256	0.03193368
2464.212266	1.785113236	0.014929252
2502.298507	1.702188545	0.024214375
2540.384748	1.584347371	0.04942289
2578.470989	1.702595408	0.02308708
2616.55723	1.601057844	0.044604403
2654.643471	1.640883615	0.034589522
**2692.729712**	**1.850538756**	**0.008755126**
2730.815953	1.853195883	0.008963489
2768.902194	1.863391509	0.008939134
2806.988435	1.640038686	0.044984899
2845.074676	1.692204722	0.03304536
2883.160917	1.729891398	0.029714302
2921.247158	1.729891398	0.029714302
2959.333399	1.679502085	0.045077266
2997.41964	1.725587137	0.040985951
3035.505881	1.75053168	0.039389894
3073.592122	1.939696431	0.019326126
3111.678363	1.876330989	0.030005828
3149.764604	1.598088372	0.125699913
3187.850845	0.9750217	0.94926715

B

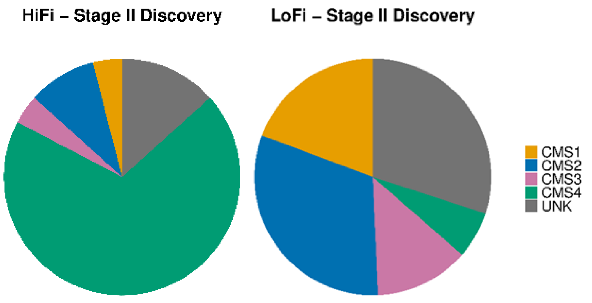

Supplementary Figure 2

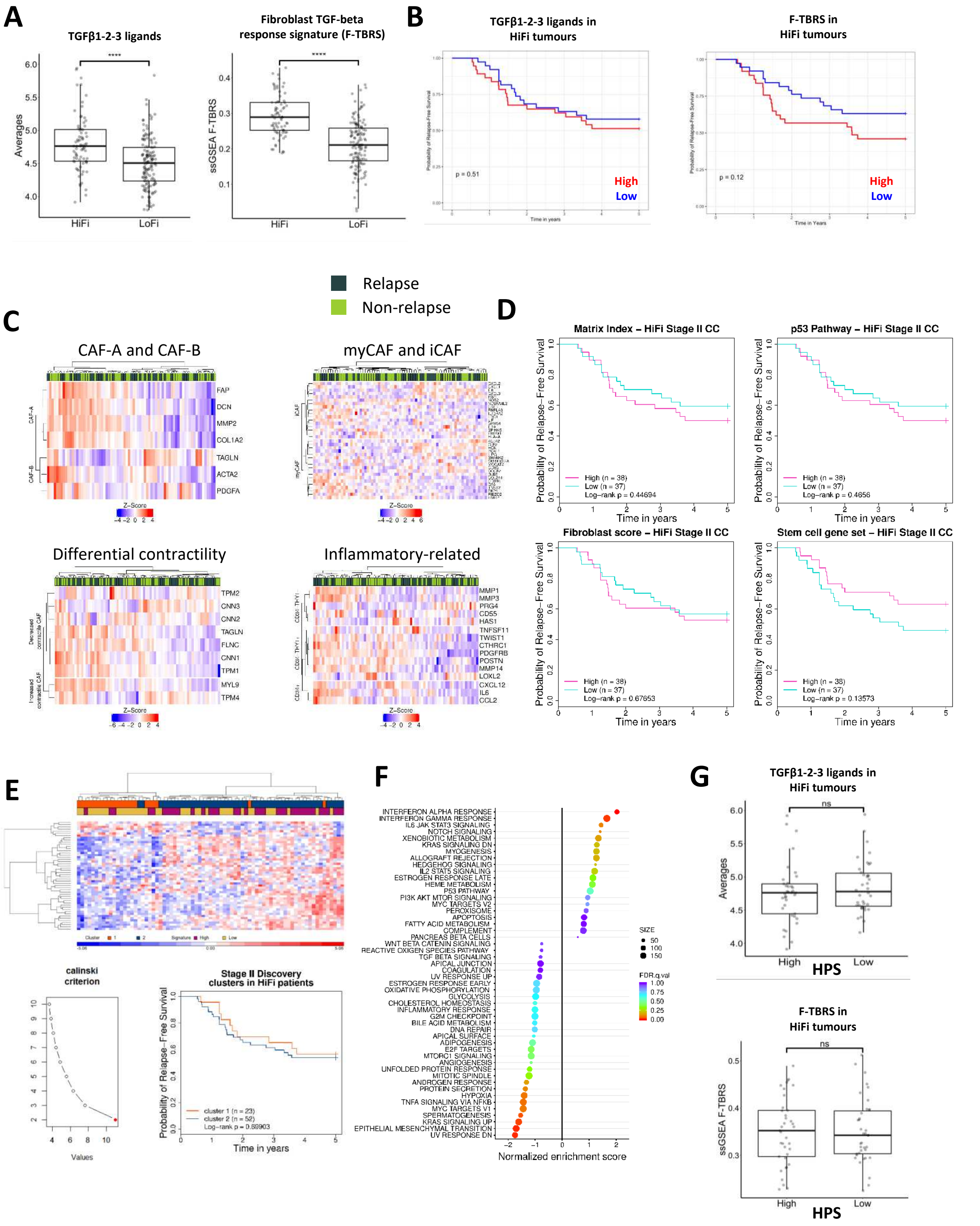

Supplementary Figure 3

A

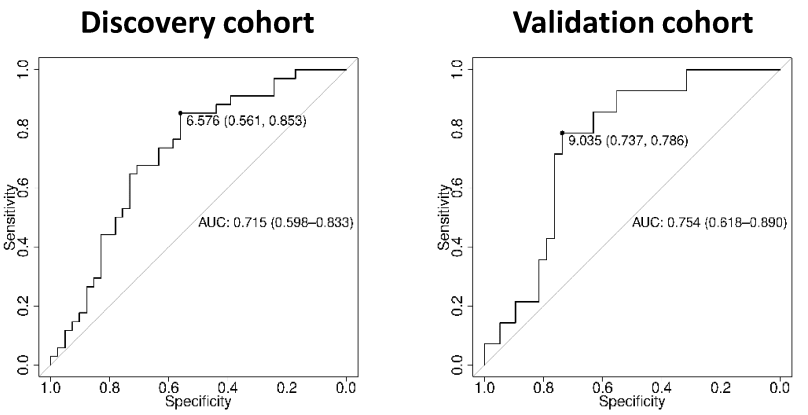

B

Discovery cohort

Method of stratification	Expression threshold	Sensitivity	Specificity
ROC	6.5755150	0.5609756	0.8529412
Median	6.4744600	0.6341463	0.6764706

Validation cohort

Method of stratification	Expression threshold	Sensitivity	Specificity
ROC	9.0351230	0.7368421	0.7857143
Median	9.1369379	0.6315789	0.8571429

Supplementary Figure 4

A

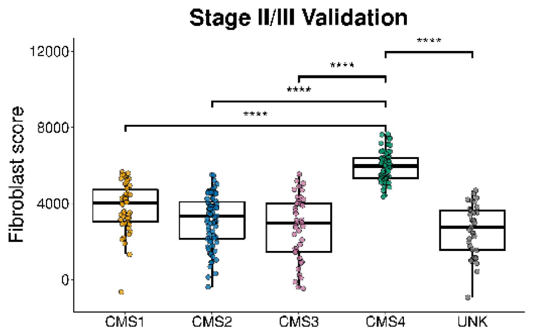

B

First author of study	Subgroup of poor prognostic mesenchymal/stem biology	Source of percentage data	Percentage of subgroup in cohort
Guinney	CMS4	Main paper text	23
Roepman	C-type	Main paper text	16
Budinska	D	Supp. Appendix S1	22.7
Sadanandam	Stem-like	Supp. Table 3	20
De Sousa E Melo	CCS3	Main paper text	27
Marisa	C4	Supp. Table S1	10.4
		**Average percentage**	**19.85**

C

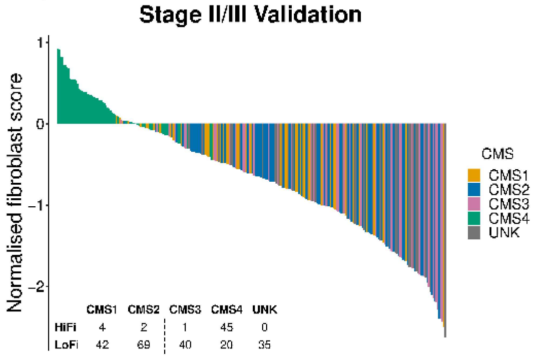

D

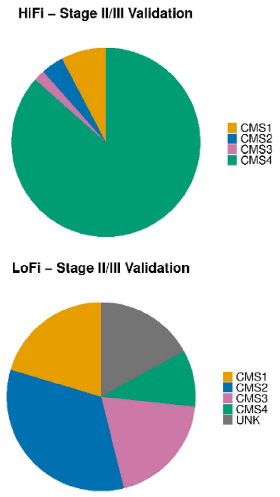

E

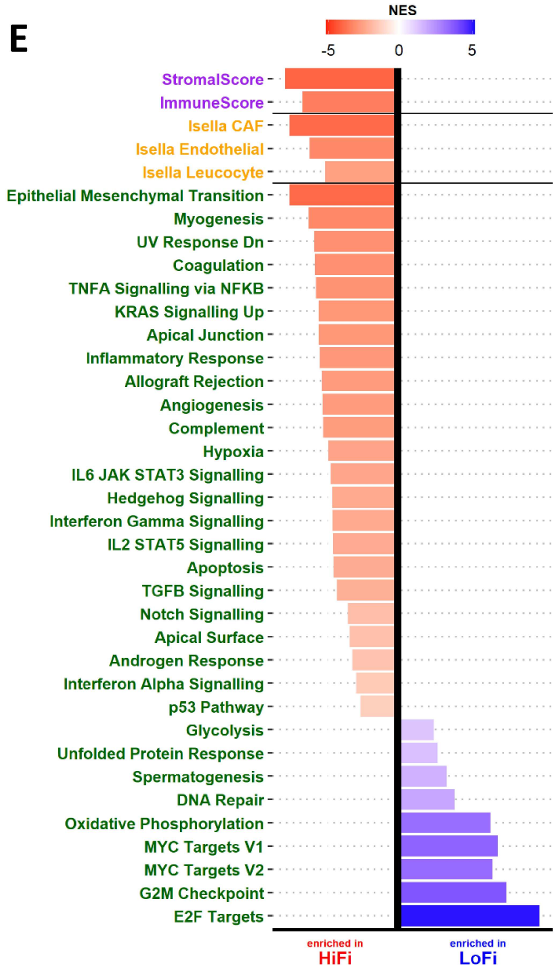

F

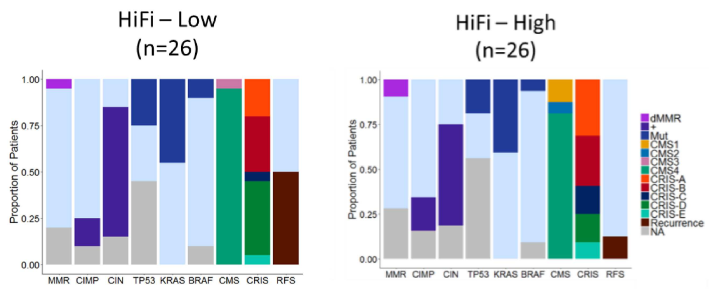

G

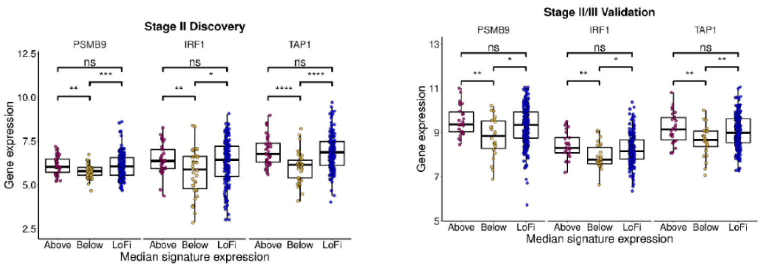

Supplementary Figure 5

A

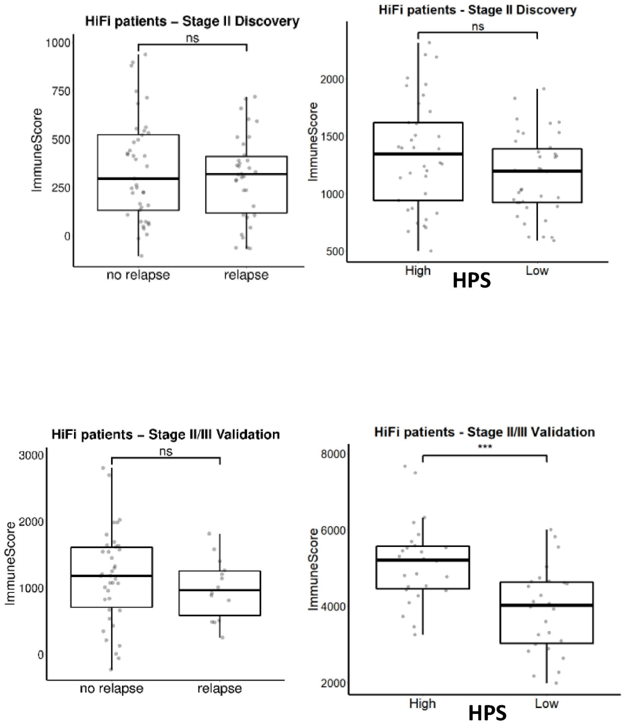

B

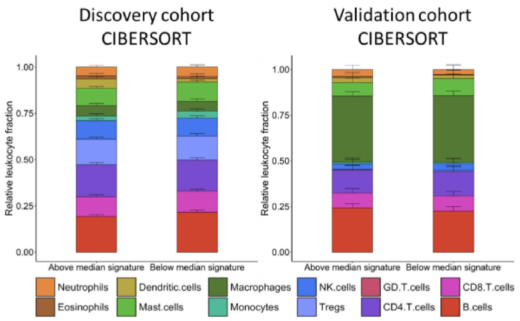

Cell population	Discovery	Validation
Neutrophils	0.74663	0.33569
Eosinophils	0.26928	0.03057
Dendritic cells	0.00076	0.51333
Mast cells	0.61458	0.27824
Macrophages	0.84487	0.85677
Monocytes	0.06597	0.22903
NK cells	0.73958	0.30186
Tregs	0.53897	0.35915
GD T cells	NA	0.18378
CD4 T cells	0.83181	0.58254
CD8 T cells	0.63046	0.90787
B cells	0.15901	0.62866

C

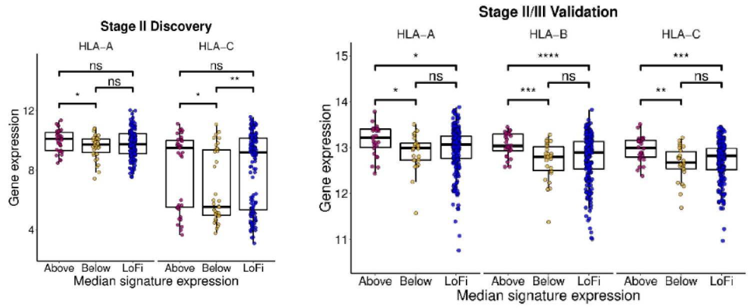

D

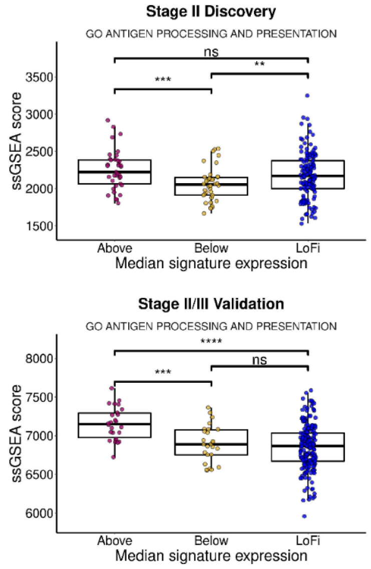

E

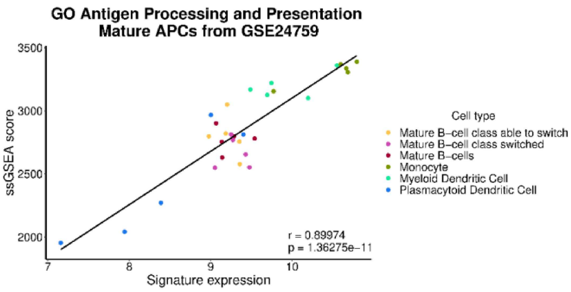

Supplementary Figure 6

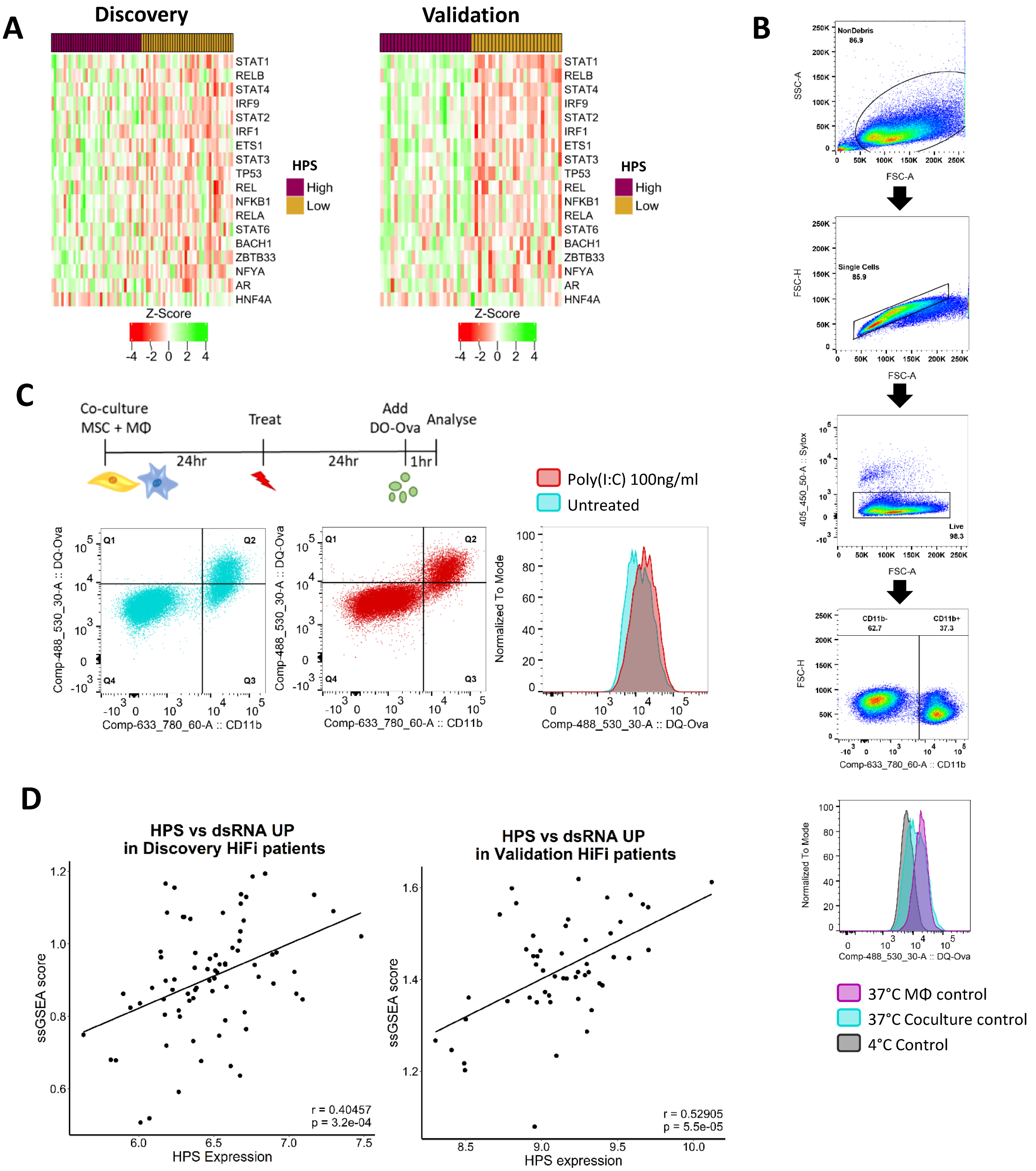

Supplementary Figure 7

A

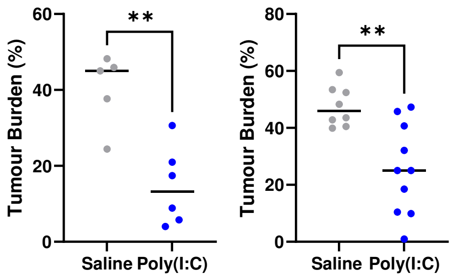

B

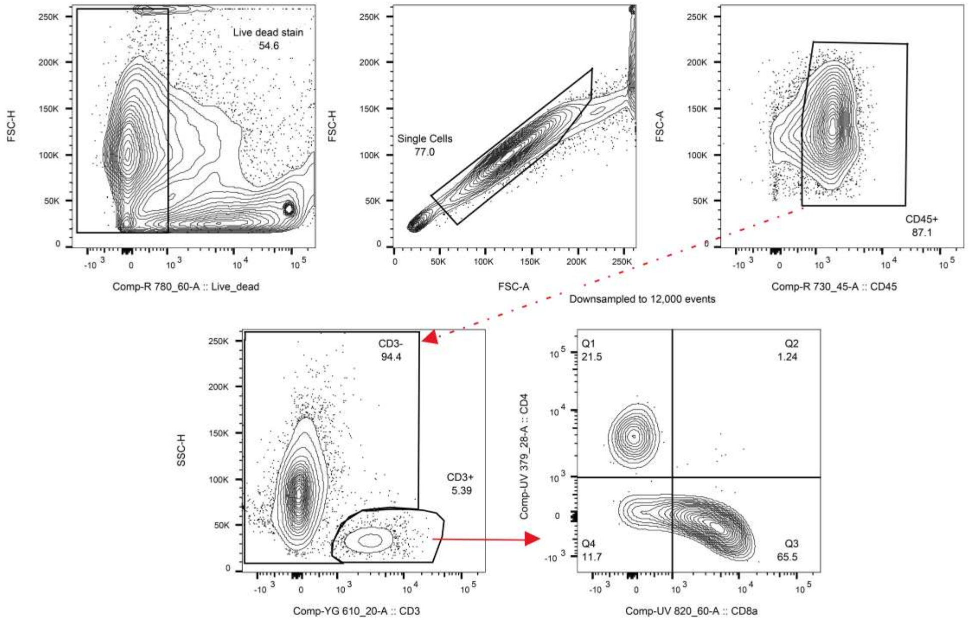

Supplementary Figure 8

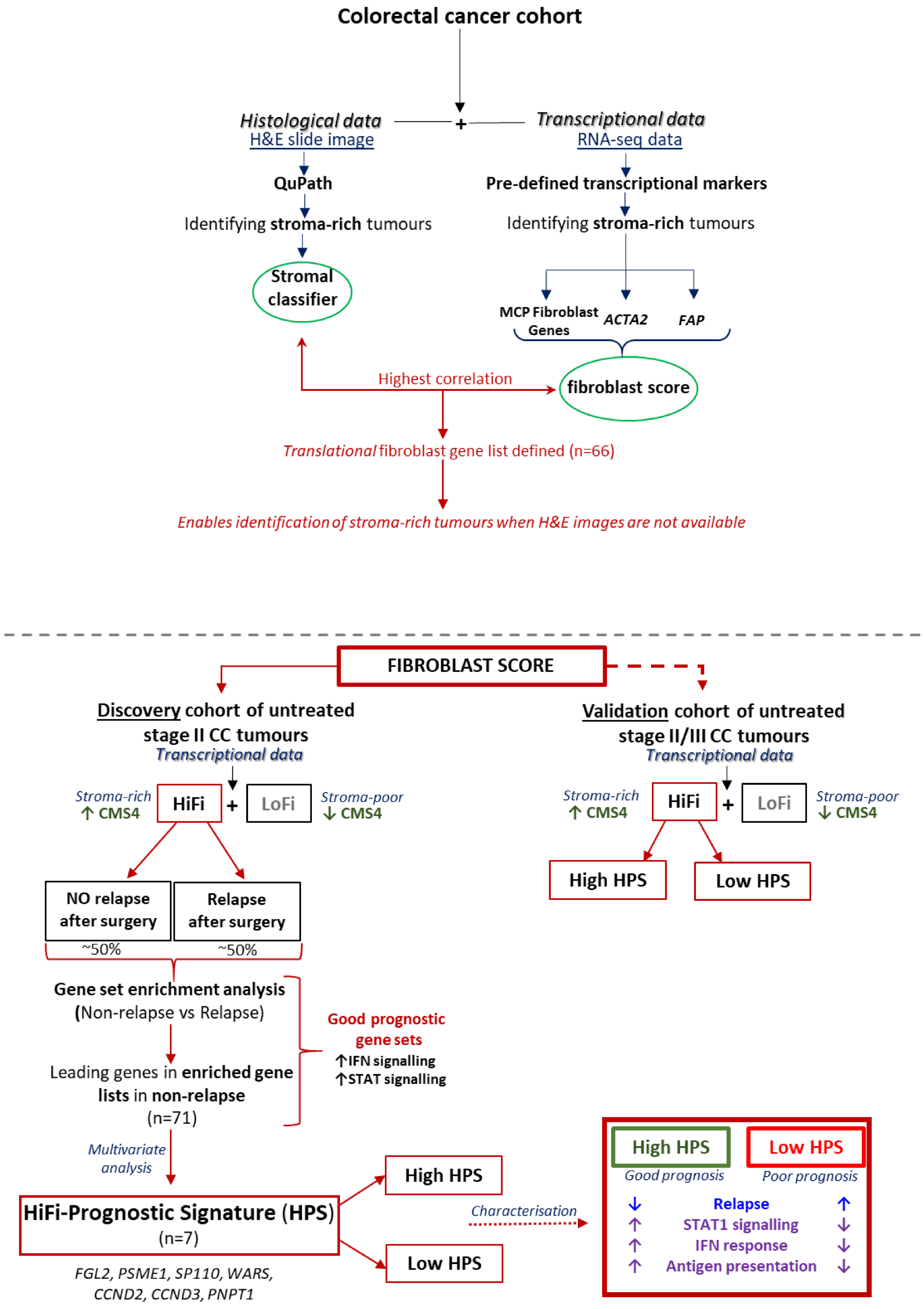

Supplementary Table 1

Custom fibroblast gene set

ACTA2	COL12A1	FAP	IGFBP5	MFGE8	PLXNA3	THY1
ADAMTS2	COL1A1	FBLN1	ITGA11	MTSS1L	PODN	TMEM119
ANGPTL2	COL3A1	FBLN5	KRTAP1-5	MXRA8	PPP1R3C	TNFRSF11B
C1R	COL6A1	FGF7	LINC01279	MYL9	PRR16	VASN
C1S	COL6A2	FIBIN	LOC100287387	PAMR1	PRRX2	VGLL3
CCDC80	COPZ2	GLT8D2	LOC100507165	PARVA	RGMB	WISP1
CD248	CREB3L1	GREM1	LOX	PCDH18	SCARF2	
CEMIP	DCN	GREM2	LPAR1	PDGFRA	STC2	
CNN1	EFEMP2	HSPB6	LRRN4CL	PDGFRB	SVEP1	
CNTN3	ELN	HSPB7	MASP1	PLAC9	TAGLN	

Supplementary Table 2

Clinical information for discovery data

Clinical Characteristic		Total cohort (n = 215) (%)	HiFi (n = 75) (%)	LoFi (n = 140) (%)
Age	Median (range)	72 (45 - 95)	72 (45 - 95)	72 (45 - 95)
Sex	Male	106 (49.3)	41 (54.7)	65 (46.3)
	Female	109 (50.7)	34 (45.3)	75 (53.6)
Recurrence (within 5 years)	Yes	73 (34.0)	34 (45.3)	39 (27.9)
	No	142 (67.4)	41 (54.7)	101 (72.1)
pT stage	3	188 (87.4)	65 (86.7)	123 (87.9)
	4	27 (12.6)	10 (13.3)	17 (12.1)
Tumor location	Proximal	136 (63.3)	44 (58.7)	92 (65.7)
	Distal	79 (36.7)	31 (41.3)	48 (34.3)
Tumor differentiation grade	Well	9 (4.2)	5 (6.7)	4 (2.9)
	Moderate	174 (80.9)	61 (81.3)	113 (80.7)
	Poor	33 (15.3)	9 (12.0)	23 (16.4)
Tumor subtype	Mucinous	37 (17.2)	13 (17.3)	24 (17.1)
	Non-mucinous	177 (82.3)	61 (81.3)	116 (82.9)
	No information	1 (0.5)	1 (1.3)	0 (0.0)
Lymphovascular invasion	Yes	36 (16.7)	10 (13.3)	26 (18.6)
	No	127 (59.1)	43 (57.3)	84 (60.0)
	No information	52 (24.2)	22 (29.3)	30 (21.4)
Number of lymph nodes assessed	Median (range)	13 (6 - 40)	11 (6 - 36)	14 (6 - 40)

HiFi patients were defined as those with ssGSEA fibroblast scores greater than the cutoff for the cohort which was generated using the findcut function.

Supplementary Table 3

Clinical information for validation data

**Clinical Characteristic**		**Total cohort (n = 258) (%)**	**HiFi (n = 52) (%)**	**LoFi (n = 206) (%)**
Age	Median (range)	73 (24 - 94)	70 (40 - 93)	73 (24 - 94)
Sex	Male	147 (57.0)	24 (46.2)	123 (59.7)
	Female	111 (43.0)	28 (53.8)	83 (40.3)
Recurrence (within 5 years)	Yes	62 (24.0)	14 (23.1)	47 (22.8)
	No	196 (76.0)	38 (73.1)	159 (77.2)
TNM stage	2	203 (78.7)	39 (75.0)	164 (79.6)
	3	55 (21.3)	13 (25.0)	42 (20.4)
Tumor location	Proximal	118 (45.7)	28 (53.8)	90 (43.7)
	Distal	140 (54.3)	24 (46.2)	116 (56.3)
MMR status	pMMR	180 (69.8)	35 (67.3)	145 (70.4)
	dMMR	47 (18.2)	4 (7.7)	43 (20.9)
	No information	31 (12.0)	13 (25.0)	18 (8.7)
CIMP status	+	52 (20.2)	9 (17.3)	43 (20.9)
	-	178 (69.0)	36 (69.2)	142 (68.9)
	No information	28 (10.9)	7 (13.5)	21 (10.2)
CIN status	+	158 (61.2)	32 (61.5)	126 (61.2)
	-	65 (25.2)	11 (21.2)	54 (26.2)
	No information	35 (13.6)	9 (17.3)	26 (12.6)
tp53 mutation	Mutant	79 (30.6)	11 (21.2)	68 (33.0)
	Wild-type	80 (31.0)	14 (26.9)	66 (32.0)
	No information	99 (38.4)	27 (51.9)	72 (35.0)
KRAS mutation	Mutant	86 (33.3)	22 (42.3)	64 (31.1)
	Wild-type	163 (63.2)	30 (57.7)	133 (64.6)
	No information	9 (3.5)	0 (0.0)	9 (4.4)
BRAF mutation	Mutant	33 (12.8)	4 (7.7)	29 (14.1)
	Wild-type	206 (79.8)	43 (82.7)	163 (79.1)
	No information	19 (7.4)	5 (9.6)	14 (6.8)

HiFi patients were defined as those within the top 20% of the ssGSEA fibroblast scores for the cohort, with the other 80% considered LoFi.

Supplementary Table 4

Poly(I:C) Signature

INSL6	IFNA10	PNP	LGALS16	PARP14	C15orf48	RAB3D
TSPO	IFNA4	IFIT3	LGALS14	NDRG1	GBP6	OASL
IFNA16	IFNA8	MLKL	XDH	CTSE	IFIH1	AGRN
IFNA2	MX1	SCT	IFNA5	GNB4	IRGM	MS4A6A
IFNA6	GLIPR2	CA13	IFNA21	SLFN12	GBP5	MS4A6E
IFNA13	SAMHD1	ZBP1	CXCL10	SLFN12L	BST2	LGALS13
IFNA7	CD40	IRF7	IFIT2	STAT2	LGALS3BP	CISH
IFNA17	CDK5R1	IFNB1	GJB2	TOR3A	CD86	AXL
CDKN2B	IL15	IL27	DDX58	ISG20	GCA	APOD
IFNA1	USP41	UBE2L6	CCND2	NT5C3A	OAS2	
IFNA14	USP18	FSCN1	C2	MNDA	DAXX	
