# Supplementary material for: Activation of innate-adaptive immune machinery by poly(I:C) exposes a therapeutic vulnerability to prevent relapse in stroma-rich colon cancer
Source: Gut. 2022 Apr 27;71(12):2502–17. doi: 10.1136/gutjnl-2021-326183 (PMC9664095; doi:10.1136/gutjnl-2021-326183)
Supplement: Supplementary data [file gutjnl-2021-326183supp003.pdf]

Supplementary Methods

Datasets and data processing

The CEL files for the discovery cohort (E-MTAB-863) were imported into Partek Genomics Suite (PGS; v6.6) and RMA normalized then log2 transformed. As Gene Set Enrichment Analysis (GSEA) uses gene-level data, the probesets on the array were collapsed. This was done by importing the normalized data into R (v3.3.2 or later) and, using the 'collapseRows' function from WGCNA (Weighted Gene Coexpression Network Analysis, RRID:SCR_003302) package (v1.61) [1], selecting the probeset with the highest mean expression per gene [2]. For the S:CORT cohorts, hematoxylin and eosin (H&E) stained slide images were also obtained. Transcriptional data for the FOCUS and Grampian cohorts was generated using the Almac Diagnostics proprietary Xcel™ GeneChip array (Affymetrix, Santa Clara, CA).

GSE41295; primary human monocytes differentiated into macrophages and treated with poly(I:C), the raw CEL files were downloaded from GEO (due to issues matching the probe IDs in the series matrix to the array annotation file) then imported into PGS and RMA normalized and log2 transformed. GSE15066: mouse macrophage cell line RAW264.7 stimulated with poly(I:C) [3]. The RAW data was downloaded from GEO, as the series matrix contained null values, and imported into R and background corrected and normalized using the limma package (LIMMA, RRID:SCR_010943; v3.30.13). GSE1925 series matrix required a log2 transformation prior to its collapse to gene-level. In the single cell cohort, dendritic cells, monocytes and macrophages were extracted and further manually curated based on graph clustering with the igraph package (v1.2.5). The dendritic population was subclassified based on the expression of highly specific marker genes (pDC: *LILRA4, IRF7, CLEC4C*; cDC1: *CLEC9A, XCR1*; cDC2: *CD1C, CLEC10A*; migDC: *CCR7, CCL17).*

Digital pathology stroma classifier

The FOCUS cohort (n = 361; FFPE resections from a randomized clinical trial)[4] and the Grampian cohort (n = 225; FFPE pre-treatment rectal cancer biopsies), were obtained from the S:CORT data portal (https://www.s-cort.org/). For FOCUS and Grampian cohorts, H&E stained whole slide images with existing pathologist annotations were scanned at 40x magnification and imported into QuPath (v2.0 milestone 9)[5]. Batch analysis was applied across all slides to detect cells (Watershed cell detection with default setup, nucleus, cell and general parameters; Intensity threshold: 0.1; Maximum background intensity: 2) within the tissue annotation. Gaussian smoothed (Radius: 25µm) was applied. Annotated regions of tumour epithelium and stroma, on a selection of cases were used to build the Random Trees object classifier (Object filter: detections (all); Features: all measurements; Classes: all classes).

Single-sample Gene Set Enrichment Analysis (ssGSEA) fibroblast classifier

The 64 fibroblast genes from MCPcounter, smooth-muscle actin (*ACTA2)* and fibroblast activation protein (*FAP)* (Supplementary table 1)[6] were used with ssGSEA to generate a fibroblast score for each patient. Discovery cohort patients were split into high-fibroblast (HiFi) or low-fibroblast (LoFi) using the 'findcut' function[7]. The 'findcut' function was used to determine the optimal cutpoint for the ssGSEA fibroblast score by identifying the cutpoint value resulting in the most significant association between LoFi and HiFi groups and relapse-free survival. For all other human transcriptional datasets, samples with the highest 20% of fibroblast scores were classified as HiFi. In GEMMs, the median split was used for the waterfall plot and top/bottom tertile was used as a surrogate for HiFi/LoFi.

HiFi cut off

We would expect that the stroma-rich group accounts from ~20% in the CRC population, which has been quite a stable and robust proportion identified in multiple histological and molecular studies. However, to ensure additional power for the Discovery work in our current study, we have utilised the cohort assembled by Almac diagnostics (published study by Kennedy et al., https://pubmed.ncbi.nlm.nih.gov/22067406/). This Discovery cohort we use in this current study was previously used for the development of the now FDA-approved ColDx/GeneFx stage II prognostic assay (https://www.almacgroup.com/diagnostics/portfolio-overview/coldx/). When this cohort was assembled, it was compared with the Surveillance, Epidemiology, and End Results (SEER) database to ensure it represented a general population with stage II colon cancer. However, although it represents the general clinical population demographics, to ensure sufficient relapse "events" for prognostic biomarker development, the tumours included were pre-selected to ensure a 2:1 ratio of "low-risk" to "high-risk". Low-risk patients were defined as those with no cancer recurrence within 5 years of primary surgery. High-risk patients were defined as those with metastatic cancer recurrence within 5 years of primary surgery. Therefore, after balancing for clinical factors and applying quality control criteria, the Discovery cohort contains 215 patients (142 low-risk and 73 high-risk patients), with a relapse rate of 34% in this cohort. By having a higher relapse rate, given the relationship between stromal content and relapse in stage II, it also contains an increased proportion of stromal tumours when compared to more general stage II or stage II/III cohorts. Discovery cohort patients were split into high-fibroblast (HiFi) or low-fibroblast (LoFi) using the 'findcut' function[7].

For these reasons, we did not use the widely-used 20% cut-off, and our optimal fibroblast score (Supplementary Figure 1A) was defined as 35% in this cohort. This enrichment is precisely why it is valuable as a Discovery cohort in our study and for the generation of prognostic classifiers and subsequent characterisation of prognostic biology. For Validation, we have utilised the well-characterised "Marisa" cohort used in the development of the CMS classification system by the CRCSC consortium (https://www.synapse.org/#!Synapse:syn2623706/wiki/67246), a cohort that isn't pre-selected in the same way as our Discovery cohort. In this case, we have identified that in line with stroma-rich proportions across all CRCSC cohorts (Supplementary Figure 4B), the consensus proportion of this stroma-rich histological group is 20%.

Gene Set Enrichment Analysis (GSEA) and Leading Edge Analysis (LEA)

GSEA using Hallmark gene sets from the Molecular Signature Database (MSigDB) with default settings (except gene_set permutations, random seed = 367707) was performed. Gene sets with an FDR < 0.25 were considered significant. LEA was used to identify genes that were shared by the leading-edge subsets of two or more significant gene sets. Following LEA (and a multivariate model) identifying the HPS, fgsea (V 1.16.0) (permutation = 1000, set.seed =121) using Hallmarks and immune-related gene lists were assessed (detailed in GitHub scripts associated with this study). Gene lists that required conversion from human to mouse or vice versa, was converted using biomaRt (V 2.46.3).

Unsupervised clustering of HiFi tumors

Transcriptional data from the HiFi patients from the discovery cohort were filtered to keep the top 50% variant genes that also had above mean expression (n = 4833). The data were then scaled using the 'scale' function from the base package. To determine the optimal number of clusters, the 'cascadeKM' function from the vegan package (v2.5-1) was used to generate a Calinski criterion for 1-

10 clusters. The optimal number of clusters was determined to be two and the 'kmeans' function from the stats package was used to split the discovery cohort into two clusters.

CMS and CRIS classifications

To classify patients into the CMS groups, the R package CMScaller (v0.99.1) was used with default settings and seed set to 367707 [8]. CRIS classifications were performed with the Nearest Template Prediction module on GenePattern (https://cloud.genepattern.org/gp; RRID:SCR_003201) with a template containing the CRIS genes from the original study [9].

Estimating relative abundance of immune and stromal cell population

Four different methods were employed to estimate the relative abundance of immune and stromal cell populations in transcriptional data from bulk tumor samples. (i) MCPcounter R package (v1.1.0), which assigns scores for eight immune and two stromal cell populations; (ii) mean expression of transcriptional signatures for cancer-associated fibroblasts, endothelial cells and leukocytes [10]. (iii) ESTIMATE (Estimation of STromal and Immune cells in MAlignant Tumor tissues using Expression data) method implemented in the R package estimate which assigns scores of general immune and stromal infiltrate [11]. (iv) the online CIBERSORT tool (https://cibersort.stanford.edu/; RRID:SCR_016955), assigning scores for 22 immune cell populations [12]. Before analysis of the CIBERSORT scores, certain populations were combined as described previously by Zemek *et al.* [13].

Transcription Factor Activity Analysis

The dorothea (V 1.2.2) and viper (V 1.24.0) packages were used to investigate transcription factor activity, with human (dorothea_hs) or mouse (dorothea_mm) versions applied depending on the dataset species.

Statistics

Pearon's Correlation: Figure 1C: Correlation matrix with histological stroma and transcriptional classifiers using Pearson's correlation. **Figure 3C:** Cumulative gene expression of *STAT1* and three of its target genes (*PSMB9, IRF1* and *TAP1*) correlated with expression of the HPS in the discovery (left) and validation cohort (right; both p<0.00001) using Pearson's correlation. **Figure 4B:** Correlation between ssGSEA scores for APP and HPS gene expression in the discovery cohort (r=0.5, p=1.4e-06) using Pearson's correlation. **Figure 4C:** Correlation between ssGSEA scores for APP and HPS gene expression in the validation cohort (r=0.6, p=1.5e-05) using Pearson's correlation. **Figure 5C:** Correlation between ssGSEA scores for viral response and HPS gene expression in the discovery cohort (r=0.6, p=1.1e-08) using Pearson's correlation **Figure 5D:** Correlation between ssGSEA scores for viral response and HPS gene expression in the validation cohort (r=0.7, p=2.4e-08) using Pearson's correlation.

T-test:

Figure 1D: CMS classification according to our fibroblast score in discovery cohort. (UNK = unknown/mixed CMS classification) using a t-test. **Figure 3D:** Boxplots of *STAT1* gene expression (left)

and protein levels (right) in HiFi patients in the CPTAC cohort according to HPS groups (High n=9 and Low n=9) using t.test**. Figure 4E, 4F:** Immune cell populations have significantly higher expression of the HPS **(4F)** and GO APP ssGSEA scores than epithelial cells and fibroblasts (both p<2.2e-16) using t-test. **Figure 4H:** APP ssGSEA scores in bone-marrow derived macrophages with either wild-type (WT), mutant (Y701F mut) or knockout (KO) *STAT1*. (n=3 for each genotype) using t.test **Figure 5A:** Activity status of key TF regulons according to HPS groups in the validation cohort (n=26 in each subgroup), p value (<0.05) determined using row t-test. **Figure 5F:** Viral response ssGSEA scores in bone-marrow derived macrophages with either wild-type (WT), mutant (Y701F mut) or knockout (KO) *STAT1*. (n=3 for each genotype) using t-test**. Figure 7A:** CMS classification according to fibroblast score of GEMM genotypes. (A=*Apc*$^{fl/fl}$, K=*Kras*$^{G12D/+}$, P=*p53*$^{fl/fl}$ and N=*Notch1Tg*/$^{+}$) using t-test.

Survival:

Figure 1F: Kaplan-Meier (KM) curve showing HiFi patients have significantly poorer relapse-free survival (RFS) than LoFi patients (log-rank p=0.00779) p-value extracted from survival package (V 3.2-13). **Figure 2E:** KM curve showed HPS has strong prognostic value in HiFi tumours based on a median split in Discovery cohort (log-rank p=0.0069) (top). KM curve showed no prognostic value in the LoFi samples in Discovery cohort (log-rank p=0.63215) (bottom). P-value was extracted from survival package (V 3.2-13). **Figure 2G:** HPS can stratify HiFi samples into two groups in the validation cohort, one with significantly poorer RFS and another with RFS even better than the LoFi patients (log-rank p=0.00113) (top). HPS has no prognostic value in the LoFi samples (log-rank p=0.46596) (bottom). P-value was extracted from survival package (V 3.2-13).

Other:

Figure 1G: Comparison of HiFi and LoFi samples revealed that previously published stromal signatures and gene sets have significantly higher expression in the HiFi samples than the LoFi (adjusted p value provided by GSEA <0.15). **Figure 6G:** Differentially expressed genes (logFC>2 and adjusted p-value <0.001) in Poly(I:C) treated vs non-treated dendritic cells creating the 'Poly(I:C) Signature'. **Figure 7G:** Digital pathology assessment of H&Es from in vivo studies demonstrates reduced liver metastasis in mice treated with poly(I:C) (n=16) compared to saline control (n=13) (Mann Whitney U test). **Figure 7H:** Flow cytometry assessment of CD3+ cell populations from liver metastases in treatment groups highlight significant elevation of CD8+ T cells alongside significant reduction in CD4+ T cells in poly(I:C) arm (n=6) compared to saline (n=5) (Mann Whitney U test).

Correlation analysis

All correlations were performed in R using the 'cor.test' function from the stats package, with the "pearson" method.

Single-sample Gene Set Enrichment Analysis (ssGSEA)

ssGSEA was also used with gene sets from the Molecular Signature Database (MSigDB; v6.0 or later) to assign TGF-β scores, using the "HALLMARK_TGF_BETA_SIGNALING" gene set from the Hallmark collection (56); p53 scores using the "HALLMARK_P53_PATHWAY" gene set from the Hallmark collection; and antigen processing and presentation scores, using the "GO_ANTIGEN_PROCESSING_AND_PRESENTATION" gene set from the C5 collection.

Analysis of previously identified prognostic factors in HiFi tumors

Signatures from four studies that have attempted to identify subgroups of fibroblasts were tested in the HiFi tumors from the discovery cohort. (A) Li *et al.* [14] who used a single-cell RNA sequencing approach and profiled cells from 11 primary colorectal tumors and normal tissue, and found within the fibroblasts from the tumors samples that two groups could be identified (CAF-A (with ECM remodeling gene expression) and CAF-B (with activated myofibroblasts signaling)). (B) Öhlund *et al.* [15] who used co-culture models of pancreatic ductal adenocarcinoma and found two reversible subtypes of CAFs existed, one with higher expression of αSMA and lower expression of IL-6 (myCAFs) and the other with lower levels of αSMA and higher expression of IL-6 (iCAFs). (C) Glenis *et al.* [16], who utilized proteomics from primary human normal fibroblasts and CAFs, and identified that CAFs had increased expression of highly contractile proteins and reduced expression of proteins associated with reduced contractility compared to normal fibroblasts. (D) Mizoguchi *et al.* [17] who looked at heterogeneity in fibroblasts using transcriptional data derived from fibroblasts from joints of patients with rheumatoid arthritis or osteoarthritis. They identified three types of fibroblasts, including a group of PDPN+CD34– THY1+ cells that were involved in immune cell recruitment and matrix invasion. Several other prognostic factors were tested in the HiFi tumors from the discovery cohort including matrix index, a measure of matrix stiffness utilizing 22 genes described by Pearce *et al.* [18]; p53 signaling, determined using ssSGEA scores as described above; our own fibroblast score, calculated using ssGSEA as described above; and the expression of *CD44*, a known marker of stemness in cancer.

ROC analysis

ROC curves were generated using the 'roc' function from the pROC R package (v1.10.0) [19]. Sensitivity and specificity values for the median cutoff were found using the 'coords' function.

Heatmaps of signature and STAT1 gene expression

Heatmaps were generated in R by first scaling the data using the 'scale' function from the base package to generate Z-scores, then clustering (using Ward's linkage and Euclidean distance) and plotting them using the 'Heatmap' function from the ComplexHeatmap package (RRID:SCR_017270; v2.0.0 or later) [20].

Differential gene expression analysis

The function 'rowttests' from the package genefilter R package (v1.56.0) was used to calculate t-tests per gene between above median and below median signature expression tumors in the discovery and validation cohorts. The p-values from these tests were adjusted using the Benjamini-Hochberg method utilized in the function 'mt.rawp2adjp' from the multtest package (v2.30.0) [21]. The final gene list consisted of genes that were significantly differentially expressed in the same direction in both cohorts.

Ingenuity Pathway Analysis (IPA)

The genes identified as differentially expressed between the HPS groups in the discovery and validation cohorts were entered into IPA (QIAGEN Inc,; RRID:SCR_008653 https://digitalinsights.qiagen.com/qiagen-ipa), a web-based application that enables the analysis of

gene expression patterns using a literature-based database, to identify upstream regulators of the differentially expressed genes and analyzed using the default settings for a core analysis [22].

Single-cell RNA-Seq data

10X sequencing of 15 distinct patients, including samples from cancer (core and tumour border) and normal tissue was performed. Cells were filtered to contain at least 300 unique genes and less than 6000 unique genes, and a mitochondrial content of less than 20%. Batch normalization with scran (v1.14) and initial cell classification with SingleR (v1.0.5) against the Human Primary Cell Atlas[23], which is a human immune cell atlas[24] and a previously published PBMC dataset[25] (https://support.10xgenomics.com/single-cell-gene-expression/datasets), were performed. After manual curation, the two main batches were integrated with MNN dimension [26] reduction for plotting.

Cell culture

CT26 cells (murine colon adenocarcinoma cells derived from Balb/c mice. RRID:CVCL_7256) were cultured in Dulbecco's Modified Eagles Medium (DMEM) High glucose with L-glutamine (Hyclone; Thermo Fisher Scientific, Waltham, MA, USA) supplemented with 10% fetal bovine serum (FBS; Sigma) and 1% penicillin/streptomycin (p/s; Gibco; Thermo Fisher Scientific, Waltham, MA, USA). RAW264.7 cells (murine macrophages derived from Balb/c mice) were cultured in DMEM high glucose, GlutaMAX™ with sodium pyruvate (Gibco) supplemented with 10% FBS and 1% p/s. Primary mesenchymal stromal cells (MSCs) were isolated from the bone marrow of Balb/c mice as previously described [27]. MSCs were cultured in Minimum Essential Medium (MEM) alpha (Gibco) supplemented with 10% FBS and 1% p/s. All cells were incubated at 37°C in 5% CO_2 and experiments were performed with MSCs between passages 8-11. Cell lines were purchased from the ATCC, confirmed mycoplasma negative (MycoAlert; Lonza; Basel, Switzerland), expanded, frozen and used within 15 passages of testing for all subsequent experiments.

MSC conditioning, co-culture and poly(I:C) treatment

Tumor-conditioned medium (TCM) was generated by seeding 1 x 10^6 CT26 cells (RRID:CVCL_7256) in a T175 flask in 25ml of medium. After 72 hours of culture, the medium was collected and spun at 1,000 RCF to remove any debris.

MSCs were seeded at a density of 9.0 x 10^4 cells per well in a 6-well plate in 2ml of culture medium. After 8 hours, the medium was removed and replaced with 40% fresh MSC medium and 60% TCM. After 48 hours, the conditioned MSCs were co-cultured with RAW264.7 cells (RRID:CVCL_0493) by seeding both cells at a density of 2.0 x 10^4 cells per well in a flat-bottom 96-well plate. A monoculture of RAW264.7 cells was also plated at a density of 2.0 x 10^4 cells per well. After 24 hours, the cultures were treated overnight with either 100ng/ml poly(I:C) (InvivoGen; San Diego, CA, USA) or RAW264.7 culture medium to act as an untreated control.

DQ-Ova flow cytometry

To assess antigen processing, we used a BODIPY FL labelled DQ™ Ovalbumin (DQ-Ova; Thermo Fischer Scientific). RAW264.7 cells (RRID:CVCL_0493), either alone or in co-culture with conditioned MSCs,

were incubated with DQ-Ova at a final concentration of 1µg/ml for one hour, washed with PBS twice, trypsinized and transferred to a v-bottom 96-well plate before being spun at 600 RCF and resuspended in FACS buffer (PBS supplemented with 2% FBS and 0.05% sodium azide). Staining was performed by incubating the cells with the CD11b antibody (Cat# 101226, RRID:AB_830642; APC/Cy7, Clone M1/70; BioLegend; San Diego, CA, USA) in FACS buffer at 4°C for 15 minutes. After two washes in FACS buffer, the cells were resuspended in FACS buffer containing SYTOX™ Blue (Thermo Fischer Scientific) for viability assessment. Samples were analyzed on a FACSCanto™II (BD Biosciences; Becton, Dickinson and Company, Franklin Lakes, NJ, USA) and analyzed using FlowJo (FlowJo, RRID:SCR_008520). Gating strategy in Supplementary Figure 6 (v10.6.1; BD Biosciences).

GEMM dataset descriptor and histology

Both genders of AKPN, APN, AP, A, KPN, KP mice were induced with a single intraperitoneal injection of 2mg tamoxifen between 7 and 14 weeks of age (n of at least 3 per genotype). Mice were sampled at clinical endpoint according to defined criteria including weight loss and/or hunching and/or anemia and/or loss of body condition. Intestinal tumours were sampled for histology and RNAseq analysis.

In vivo tissue processing by flow cytometry

The tissues for flow cytometry analysis of in vivo samples were processed the following way: The blood samples were mixed with an erythrocyte lysis buffer (For 1L: 8.29g NH_4Cl, Sigma-Aldrich, Cat#A9434; 1.0g $KHCO_3$, Sigma-Aldrich, Cat#60339; 37.2mg Na_2EDTA, Sigma-Aldrich, Cat#E5134; 1L dH_2O, adjusting pH to 7.2-7.4). The blood cells were incubated for 10 minutes at 4°C. The liver metastasis samples were dissected and digested using the Mouse Tumour Dissociation Kit (Miltenyi Biotec, 130-096-730) and the GentleMACS Octo Dissociator (Miltenyi Biotec, 130-095-937), following the manufacturer's instructions for these tumours.

Cells from both tissue samples were passed through a 70µm cell strainer, washed with RPMI 1640, 10% fetal bovine serum, 2mM EDTA, and spun down at 400g, 4°C for 5 minutes. The cells were washed with PBS and spun down at 400g, 4°C for 5 minutes. After discarding the supernatant, the cells were stained with 50 µL of the Live/Dead NIR staining kit, at 1:1000 dilution in PBS, for 20min at 4°C in the dark. After that, the cells were washed with PBS 1% BSA and spun down at 400g, 4°C for 5 minutes. After discarding the supernatant, the cells were resuspended in 25µL of FcBlock (BD Pharmingen, 553141) 1:200 in PBS 1% BSA, and incubated for 10 minutes on ice in the dark. Then, 25µL of the antibody staining mix at 2x concentration were added (see table below for antibody details).

Marker	Fluorochrome	Company	Catalog no.
**MHCI**	PerCP/Cy5.5	Biolegend	114620
**CD86**	FITC	Biolegend	105110
**CSF1R**	BV786	BD Biosciences	750888
**CD103**	BV711	Biolegend	121435
**CD11b**	BV650	Biolegend	101259
**CD11c**	BV605	Biolegend	117334

**F4/80**	BV510	Biolegend	123135
**MHCII**	BV421	Biolegend	107632
**CD8a**	BUV805	BD Biosciences	612898
**CD69**	BUV737	BD Biosciences	612793
**CD4**	BUV395	BD Biosciences	563790
**CD45**	AF700	Biolegend	103128
**Ly6G**	AF647	Biolegend	127610
**CD64**	PE/Cy7	Biolegend	139314
**CD3**	PE/Dazzle594	Biolegend	100246
**CD26**	PE	Biolegend	137804

The cells were incubated for 30 minutes on ice in the dark. The cells were washed with PBS and re-suspended in 50µL PBS. The cells were fixed adding 50µL 4% PFA in PBS and incubated for 15 minutes at room temperature in the dark. The cells were washed with PBS two times spinning down at 400g for 5 minutes. They were resuspended in 400µL PBS for data acquisition in the flow cytometer. Countbright beads (ThermoFisher Scientific, C36950) were added for absolute cell quantifications at this step.

For initial QC, immune cells populations were identified in a BD LSRFortessa (BD Biosciences) using the FACSDiva software, with the following gating strategy: cells in a FSC-A and SSC-A plot; doublet discrimination by discrepancy between FSC-A and FSC-H signals; Live/Dead- live cells; CD45+ for all immune cells. The T-cells were identified as CD3+, then looked for CD69, CD4 and CD8 markers. Neutrophils where identified as CD3-CD11b+Ly6G+. Eosinophils as CD3-SSChiLy6G-. Monocytes (in blood only) as CD11b+Ly6G-CSF1R+. Macrophages (in liver metastatic tissue only) as CD11b+Ly6G-F4/80+CD64+. Dendritic cells were identified as CD3-Ly6G-MHCII+CD11c+F4/80-CD64-, then looked for MHCI, CD26, CD86, CD11b and CD103. The flow cytometry data were analysed using FlowJo v10.7.2.

Patient and Public Involvement: Patients or the public were not involved in the design, or conduct, or reporting, or dissemination plans of our research.

Data availability: RRIDs (research resource identifiers) are used to identify resource within the Methods section. Any data used in this study that are not already publically-available will be shared, please contact corresponding author (p.dunne@qub.ac.uk). In addition, code to reproduce all results will be deposited on our lab website (www.dunne-lab.com).

Supplementary Method References:

1. Langfelder P, Horvath S. WGCNA: an R package for weighted correlation network analysis. *BMC Bioinformatics* 2008;9(1):559. doi: 10.1186/1471-2105-9-559
2. Ding L, Ley TJ, Larson DE, et al. Clonal evolution in relapsed acute myeloid leukaemia revealed by whole-genome sequencing. *Nature* 2012;481(7382):506-10. doi: 10.1038/nature10738; 10.1038/nature10738
3. Yang IV, Jiang W, Rutledge HR, et al. Identification of novel innate immune genes by transcriptional profiling of macrophages stimulated with TLR ligands. *Molecular Immunology* 2011;48(15):1886-95. doi: https://doi.org/10.1016/j.molimm.2011.05.015
4. Seymour MT, Maughan TS, Ledermann JA, et al. Different strategies of sequential and combination chemotherapy for patients with poor prognosis advanced colorectal cancer (MRC FOCUS): a randomised controlled trial. *The Lancet* 2007;370(9582):143-52. doi: 10.1016/S0140-6736(07)61087-3
5. Bankhead P, Loughrey MB, Fernández JA, et al. QuPath: Open source software for digital pathology image analysis. *Scientific Reports* 2017;7(1):16878. doi: 10.1038/s41598-017-17204-5
6. Becht E, Giraldo NA, Lacroix L, et al. Estimating the population abundance of tissue-infiltrating immune and stromal cell populations using gene expression. *Genome biology* 2016;17(1):218. doi: 10.1186/s13059-016-1070-5
7. Chang C, Hsieh M-K, Chang W-Y, et al. Determining the optimal number and location of cutoff points with application to data of cervical cancer. *PLOS ONE* 2017;12(4):e0176231.
8. Eide PW, Bruun J, Lothe RA, et al. CMScaller: an R package for consensus molecular subtyping of colorectal cancer pre-clinical models. *Scientific Reports* 2017;7(1):16618. doi: 10.1038/s41598-017-16747-x
9. Alderdice M, Richman SD, Gollins S, et al. Prospective patient stratification into robust cancer-cell intrinsic subtypes from colorectal cancer biopsies. *The Journal of pathology* 2018;245(1):19-28. doi: 10.1002/path.5051 [doi]
10. Isella C, Terrasi A, Bellomo SE, et al. Stromal contribution to the colorectal cancer transcriptome. *Nature genetics* 2015;47(4):312–19. doi: 10.1038/ng.3224
11. Yoshihara K, Shahmoradgoli M, Martinez E, et al. Inferring tumour purity and stromal and immune cell admixture from expression data. *Nature communications* 2013;4:2612. doi: 10.1038/ncomms3612 [doi]
12. Gentles AJ, Newman AM, Liu CL, et al. The prognostic landscape of genes and infiltrating immune cells across human cancers. *Nature medicine* 2015;21(8):938-45. doi: 10.1038/nm.3909 [doi]
13. Zemek RM, De Jong E, Chin WL, et al. Sensitization to immune checkpoint blockade through activation of a STAT1/NK axis in the tumor microenvironment. *Science Translational Medicine* 2019;11(501):eaav7816. doi: 10.1126/scitranslmed.aav7816
14. QuPath: Open source software for digital pathology image analysis. *bioRxiv* 2017 doi: 10.1101/099796 "A1 Bankhead, Peter" "A1 Loughrey, Maurice B" "A1 Fernández, José A" "A1 Dombrowski, Yvonne" "A1 McArt, Darragh G" "A1 Dunne, Philip D" "A1 McQuaid, Stephen" "A1 Gray, Ronan T" "A1 Murray, Liam J" "A1 Coleman, Helen G" "A1 James(TRUNCATED)
15. Öhlund D, Handly-Santana A, Biffi G, et al. Distinct populations of inflammatory fibroblasts and myofibroblasts in pancreatic cancer. *The Journal of experimental medicine* 2017 doi: 10.1084/jem.20162024
16. Glentis A, Oertle P, Mariani P, et al. Cancer-associated fibroblasts induce metalloprotease-independent cancer cell invasion of the basement membrane. *Nature Communications* 2017;8(1):924. doi: 10.1038/s41467-017-00985-8
17. Mizoguchi F, Slowikowski K, Wei K, et al. Functionally distinct disease-associated fibroblast subsets in rheumatoid arthritis. *Nature Communications* 2018;9(1):789. doi: 10.1038/s41467-018-02892-y

18. Pearce OMT, Delaine-Smith RM, Maniati E, et al. Deconstruction of a Metastatic Tumor Microenvironment Reveals a Common Matrix Response in Human Cancers. *Cancer Discov* 2018;8(3):304-19. doi: 10.1158/2159-8290.CD-17-0284 [published Online First: 2017/12/01]
19. Jansen FH, van Rijswijk A, Teubel W, et al. Profiling of Antibody Production against Xenograft-released Proteins by Protein Microarrays Discovers Prostate Cancer Markers. *Journal of proteome research* 2012;11(2):728-35. doi: 10.1021/pr2006473
20. Gu Z, Eils R, Schlesner M. Complex heatmaps reveal patterns and correlations in multidimensional genomic data. *Bioinformatics* 2016;32(18):2847-49. doi: 10.1093/bioinformatics/btw313
21. Pollard KS, Dudoit S, van der Laan MJ. Multiple Testing Procedures: the multtest Package and Applications to Genomics. In: Gentleman R, Carey VJ, Huber W, et al., eds. Bioinformatics and Computational Biology Solutions Using R and Bioconductor. New York, NY: Springer New York 2005:249-71.
22. Krämer A, Green J, Pollard J, Jr, et al. Causal analysis approaches in Ingenuity Pathway Analysis. *Bioinformatics* 2013;30(4):523-30. doi: 10.1093/bioinformatics/btt703
23. Mabbott NA, Baillie JK, Brown H, et al. An expression atlas of human primary cells: inference of gene function from coexpression networks. *BMC Genomics* 2013;14(1):632. doi: 10.1186/1471-2164-14-632
24. Monaco G, Lee B, Xu W, et al. RNA-Seq Signatures Normalized by mRNA Abundance Allow Absolute Deconvolution of Human Immune Cell Types. *Cell Reports* 2019;26(6):1627-40.e7. doi: 10.1016/j.celrep.2019.01.041
25. Zheng GXY, Terry JM, Belgrader P, et al. Massively parallel digital transcriptional profiling of single cells. *Nature Communications* 2017;8(1):14049. doi: 10.1038/ncomms14049
26. Haghverdi L, Lun ATL, Morgan MD, et al. Batch effects in single-cell RNA-sequencing data are corrected by matching mutual nearest neighbors. *Nature Biotechnology* 2018;36(5):421-27. doi: 10.1038/nbt.4091
27. O'Malley G, Treacy O, Lynch K, et al. Stromal cell PD-L1 inhibits CD8+ T-cell antitumor immune responses and promotes colon cancer. *Cancer Immunology Research* 2018:canimm.0443.2017. doi: 10.1158/2326-6066.Cir-17-0443
