# Supplementary material for: Activation of innate-adaptive immune machinery by poly(I:C) exposes a therapeutic vulnerability to prevent relapse in stroma-rich colon cancer
Source: Gut. 2022 Apr 27;71(12):2502–17. doi: 10.1136/gutjnl-2021-326183 (PMC9664095; doi:10.1136/gutjnl-2021-326183)
Supplement: Supplementary data [file gutjnl-2021-326183supp002.pdf]

Supplementary Figure Legends

Supplementary Figure 1.

A Tested cutoff points of fibroblast scores using "findcut" in the discovery cohort and their corresponding hazard ratio (HR1) and p-values (P1), with the chosen cutoff shown in red. **B** Pie charts of the CMS classification in the discovery cohort (n=215 patients) in the HiFi patients (n=75) (left) and LoFi patients (n=140) (right).

Supplementary Figure 2.

A Assessment of previously published TGF-β signalling signatures are significantly associated with HiFi and LoFi groups in our discovery cohort. **B** Kaplan-Meier (KM) plots showing no significant difference between HiFi patients with high or low scores (median split) for the TGF-β signalling signatures**. C** Heatmaps showing previous cancer-associated fibroblast (CAF) classification. Li *et al.* CAF-A and CAF-B (top left), Öhlund *et al.* myCAF and iCAF (top right), Glentis *et al.* CAF with increased contractility and decreased contractility (bottom left) and Mizoguchi *et al.* $CD34^{-}THY1^{+}$, $CD34^{-}THY1^{-}$ and $CD34^{+}$ CAF (bottom right); none of the classifiers clustered the patients in the discovery cohort (n=215 patients) by relapse (dark green = relapse, light green = no relapse). **D** Kaplan-Meier (KM) plots showing no significant difference between HiFi patients with high or low scores (median split) for matrix index (p=0.44694), p53 signaling determined using the Hallmark gene set from MSigDB (p=0.4656), fibroblast scores determined using the custom fibroblast gene set (p=0.67653) or an expression signature of stem markers (p=0.13573). **E** Unsupervised clustering was performed in the HiFi patients from the discovery cohort. Calinski-Harabasz criterion was used to determine the optimal number of clusters (bottom left) which was 2. The heatmap (top) compares the unsupervised clusters (using k-means clustering; labelled with orange and navy) to the HiFi-specific prognostic signature (HPS) above and below median patients (labelled purple and yellow). KM of the HiFi patients (bottom right) split based on the two clusters identified using k-means clustering, showing no significant difference in RFS (log-rank p=0.69903). **F** Full GSEA results from the discovery cohort comparing HiFi patients who experienced relapse after surgery vs those who did not. There are 9 gene sets (UV RESPONSE DN, EPITHELIAL MESENCHYMAL TRANSITION, KRAS SIGNALING UP, SPERMATOGENESIS, MYC TAGRETS V1, TNFA SIGNALING VIA NFKB, HYPOXIA, PROTEIN SECRETION and ANDROGEN RESPONSE) significantly enriched (FDR<0.25) in the poor prognosis (relapse after surgery) patients and 10 gene sets significantly enriched in the good prognosis (no relapse after surgery) patients (INTERFERON ALPHA REPONSE,INTERFERON GAMMA REPONSE, IL6 JAK STAT3 SIGNALING, NOTCH SIGNALING, XENOBIOTIC METABOLISM, KRAS SIGNALING DN, MYOGENESIS, ALLOGRAFT REJECTION, HEDGEHOG SIGNALING and IL2 STAT5 SIGNALING). **G** Assessment of TGF-β signalling signatures reveals no association with our new HiFi-specific prognostic signature (HPS).

Supplementary Figure 3.

A. Receiver operating characteristic (ROC) curves for the HPS obtained when predicting relapse in HiFi patients from the discovery (n=75) (left) and validation cohorts (n=52) (right). The optimal cutoff which maximized the area under curve (AUC) for each cohort are shown, along with the maximum AUC value with 95% confidence intervals. **B** Comparison of the HPS cutoffs determined using either ROC or median HPS expression in the discovery (upper) and validation cohorts (lower), showing the sensitivity and specificity obtained using each cutoff.

Supplementary Figure 4.

A CMS classification according to fibroblast score. **B** Percentage of the stroma-rich, mesenchymal/stem subgroups in previously published colorectal cancer (CRC) subtyping studies. **C** Waterfall plot of fibroblast scores indicating CMS classification. High-fibroblast (HiFi) n=52 and low-fibroblast (LoFi) n=206 . **D** Pie charts of the CMS classifications in the validation cohort in the HiFi patients (n=52) (left) and LoFi patients (n=206) (right). **E** Comparison of HiFi and LoFi samples revealed that previously published stromal signatures and gene sets have significantly higher expression in the HiFi samples than the LoFi (adjusted p-value<0.15). **F** Comparison of the mismatch repair status, *CIMP, CIN*, *TP53*, *KRAS* and *BRAF* mutations, CMS and CRIS classifications and relapse status of HiFi patients from the validation cohort with low (below median) or high (above median) expression of the HPS. **G** The expression of the three *STAT1* target genes was significantly higher in the above median signature samples (n=37) compared to the below median samples (n=38) in the discovery cohort (upper; all p<0.01) and in the validation cohort (n=26 in each subgroup) (lower; all p<0.01). (Reference for all figures: ns = p>0.05, * = p≤0.05, ** = p≤0.01, *** = p≤0.001, **** = p≤0.0001).

Supplementary Figure 5.

A ImmuneScore derived from ESTIMATE in discovery (relapse=34, non-relapse=41) and validation cohorts (relapse=14, non-relapse=38) according to relapse status (left). ImmuneScore derived from ESTIMATE in discovery and validation cohorts according to HPS subgroups (right). **B** Relative CIBERSORT scores for immune cell populations in HiFi patients with above and below median HPS in the discovery (left) and validation cohort (right). The table shows each immune cell population and whether there was a significant difference in the scores between signature groups in each cohort. **C** The expression of the major histocompatibility class I genes *HLA-A* and *HLA-C* was significantly higher in the above median HPS samples (n=37) in the discovery cohort compared to the below median samples (n=38) (upper; both p< 0.05; note *HLA-B* was not on the microarray used for this cohort) and *HLA-A*, *HLA-B* and *HLA-C* were significantly higher in the above median HPS samples in the validation cohort (n=26) compared to the below median samples (n=26) (lower; all p<0.05). **D** HiFi patients with above median HPS expression had significantly higher GO APP ssGSEA scores compared to those with below median HPS expression in the discovery cohort (left; p<0.001) and in the validation cohort (right; p<0.001). **E** Correlation between ssGSEA scores for APP and HPS gene expression in purified mature antigen presenting cells. (Reference for all figures: ns = p>0.05, * = p≤0.05, ** = p≤0.01, *** = p≤0.001, **** = p≤0.0001).

Supplementary Figure 6.

A Heatmap showing the transcription factor activity scores according to HPS groups in the discovery (High=37, Low=38) and validation cohorts (n=26 for each subgroup). **B** Gating strategy and **C** experimental set up for flow cytometry analysis of macrophage mediated antigen uptake and processing of fluorescently labelled ovalbumin protein (DQ-Ova) with or without co-culture with tumor conditioned MSCs. **D** Correlation between ssGSEA scores for dsRNA and HPS gene expression in the discovery and validation cohorts (Pearson's correlation).

Supplementary Figure 7.

A Digital pathology assessment of H&Es from individual *in vivo* studies demonstrates reduced liver metastasis in mice treated with poly(I:C) compared to saline control. **B** Gating strategy; Cells were gated based on live cell status from live/dead stain, single cells and CD45+ cells selected. From here, data was downsampled to 12,000 events per sample and CD3+CD4+ and CD3+CD8+ cells were identified.

Supplementary Figure 8.

BMJ Publishing Group Limited (BMJ) disclaims all liability and responsibility arising from any reliance placed on this supplemental material which has been supplied by the author(s)

Schematic overview of initial fibroblast stratification, followed by identification and characterization of HiFi-Prognostic Signature (HPS).
