# Supplementary figures and images for: Activation of innate-adaptive immune machinery by poly(I:C) exposes a therapeutic vulnerability to prevent relapse in stroma-rich colon cancer
Source: Gut. 2022 Apr 27;71(12):2502–17. doi: 10.1136/gutjnl-2021-326183 (PMC9664095; doi:10.1136/gutjnl-2021-326183)

Graphical summary

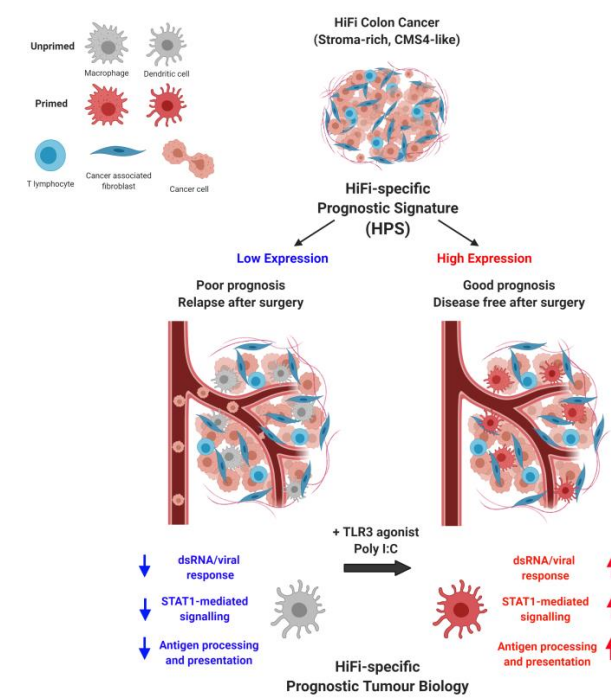

Supplement: Supplementary data [file gutjnl-2021-326183supp004.pdf]
